# Thermodynamic Study of Amine-Based Deep Eutectic Solvents with H_2_O

**DOI:** 10.1021/acs.jced.5c00695

**Published:** 2026-02-06

**Authors:** Zhida Zuo, Yusi Shen, Linghong Lu, Yudan Zhu, Xiaohua Lu, Xiaoyan Ji

**Affiliations:** † Division of Energy Science/Energy Engineering, Luleå University of Technology, 97187 Luleå, Sweden; ‡ State Key Laboratory of Materials-Oriented Chemical Engineering, 91599 Nanjing Tech University, Nanjing 210009, P. R. China; § Suzhou Laboratory, Suzhou 215125, P. R. China

## Abstract

The densities and viscosities of ([C_*n*_mimCl]­[MEA] + H_2_O) systems (*n* = 2, 4, 6) were measured over 288.15–323.15 K, and the enthalpies of mixing were determined at 298.15 and 308.15 K. Density data showed nonmonotonic composition dependence with extrema near *x*_DES_ ≈ 0.4–0.5, and negative excess molar volumes indicated enhanced molecular packing. Viscosity increased sharply at low DES content and more gradually toward pure DESs, exhibiting S-shape deviation profiles that reflect structural rearrangements in both DES-rich and H_2_O-rich regions. Negative enthalpies of mixing confirmed exothermic mixing due to strong DES–H_2_O interactions, and the data were well correlated by the NRTL model. The coincidence of extrema in density and enthalpy of mixing, together with viscosity transitions, suggests the formation of complexes among the DES constituents and H_2_O, probably leading to compact ternary DES-like microstructures. Further comparative analysis revealed that H_2_O addition reorganizes the hydrogen-bond network, forming extensive MEA–H_2_O associations. These findings offer molecular-level insights for the rational design of DES-based solvents for CO_2_ capture and related separation processes.

## Graphical abstract

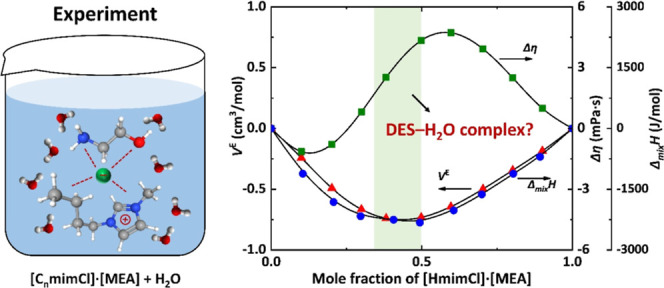

## 1 Introduction

Over the past decades, carbon capture and storage (CCS) technologies have been recognized as one of the most promising strategies for mitigating greenhouse gas emissions.^1^ Aqueous amine solution, particularly monoethanolamine (MEA), has been widely employed for CO_2_ absorption owing to its high absorption capacity, low viscosity, and relatively low cost. Nevertheless, several inherent drawbacks, including high energy usage during solvent regeneration, equipment corrosion, and solvent volatility, significantly limit their large-scale industrial application.^2^

Ionic liquids (ILs) and their analogues, deep eutectic solvents (DESs), have emerged as green and promising alternatives to conventional amine-based solvents for CO_2_ capture.^3^ Both systems have been extensively studied due to their shared advantageous properties, including negligible vapor pressure, high thermal stability, and tunable physicochemical characteristics.^4–6^ Despite these similarities, important differences distinguish ILs from DESs. ILs are composed of an anion and a cation and are typically synthesized through chemical reactions that generate byproducts and require multiple purification steps. In contrast, DES are formed through hydrogen bonding interactions between a hydrogen-bond acceptor (HBA) and a hydrogen-bond donor (HBD) and can be readily prepared by simple mixing of inexpensive, readily available components. This straightforward and low-waste preparation provides significant economic and practical advantages, making DESs particularly attractive for a variety of industrial applications,^4–6^ including sustainable CO_2_ capture.^3,7^ In particular, amine-based DESs, where MEA or other amines (e.g., methyl-diethanolamine (MDEA) and ethylenediamine (EDA)) serve as HBDs, have shown notable CO_2_ absorption capacities and the potential for reduced regeneration energy and corrosivity.^8–11^

A major practical limitation of many amine-based DESs is their relatively high viscosity, which reduces CO_2_ diffusivity and mass transfer rates,^12–14^ thereby lowering absorption efficiency and requiring larger, more costly capture columns. In addition, increased pump power and reduced heat-transfer efficiency raise the overall energy demand of the CO_2_ capture process.^15^ Adding molecular cosolvents such as water (H_2_O) can reduce the overall viscosity of the mixtures; however, it also affects other key physicochemical properties, including density and enthalpy.^5,16,17^ Several studies have reported nonmonotonic variations in density and viscosity with increasing water content in amine-based DES–H_2_O mixtures.^14,18–20^ These phenomena have been attributed to microstructure rearrangements and competitive hydrogen-bonding (H-bond) interactions between DES components and water. Theoretical studies suggest that water can integrate into the DES structures, compete with HBDs, and even act as a bridging species;^21–24^ in some cases, water addition promotes the formation of new ternary, DES-like structures.^25,26^ Despite these insights, the current understanding remains insufficient to elucidate nonmonotonic behavior observed in amine-based DES–H_2_O systems. To advance both fundamental insights and practical application, systematic studies into their physicochemical properties are essential.

Previous studies have demonstrated that amine-based DESs incorporating imidazolium-based ILs as HBAs exhibit favorable CO_2_ absorption performance. For example, Cao et al.^27^ synthesized DESs using imidazolium chloride and MEA, while Shukla et al.^12,28,29^ reported DESs composed of imidazolium chloride and EDA; both systems showed promising CO_2_ uptake. Nevertheless, several recently developed amine-based DESs employing non-IL HBAs have been reported to achieve higher CO_2_ absorption capacities than those based on imidazolium ILs,^8–11,13,14,29–32^ whereas the latter remain of particular scientific interest. This interest arises from the extensive literature on the properties of imidazolium-based ILs and their mixtures with water,^33–39^ which provides a well-established framework for interpreting DES–H_2_O behavior and assessing preferential component–water interactions. Moreover, the use of imidazolium-based ILs as HBAs enables comparison between ILs and DESs,^40,41^ facilitating the identification of DES-specific mixing phenomenon. Notably, DESs composed of imidazolium chloride and MEA can form homogeneous liquid phases over a wide temperature range and exhibit good thermal stability and reproducible preparation.^27^ Accordingly, these systems are employed as model systems to probe DES-specific mixing behavior in the presence of water.

Motivated by these considerations and the lack of systematic experimental data, we examined a series of DESs composed of 1-alkyl-3-methylimidazolium chloride ([C_*n*_mim]­Cl, *n* = 2, 4, 6) as the HBA and MEA as the HBD in a 1:4 molar ratio, i.e., [EmimCl]­[MEA] (1:4), [BmimCl]­[MEA] (1:4), and [HmimCl]­[MEA] (1:4). To date, only the density of neat [BmimCl]­[MEA] (1:4) has been reported,^27^ while other properties, especially for DES–H_2_O mixtures, remain limited. Therefore, we reported densities and viscosities of these DESs mixed with water over 288.15–323.15 K, together with enthalpies of mixing at 298.15 and 308.15 K. Excess molar volumes and viscosity deviations were derived to elucidate nonideal mixing behavior, with enthalpies of mixing providing a complementary thermodynamic insight. In addition, binary ([Hmim]Cl + H_2_O) and (MEA + H_2_O) systems were examined to distinguish H_2_O–HBA and H_2_O–HBD interactions and to assess whether the observed behaviors are intrinsic to the DES or arise from preferential DES component–water interactions.

## 2 Experimental Methods

### 2.1 Chemicals

The specifications of the used chemicals are summarized in Table 1. The ILs ([Emim]­Cl, [Bmim]­Cl, and [Hmim]­Cl) were dried under vacuum (343.15 K, 1 × 10^3^ Pa) for 4 days to remove residual moisture. MEA was used as received without further purification. Ultrapure water (5.5 × 10^–6^ S/m) was obtained from an ultrapure water machine (EPED-PLUS-E2).

**Table 1:** Chemicals Used in This Work

chemical name	molecular formula	CAS number	molar mass (kg·mol^–1^)	initial purity (mass fraction)	source
[Emim]Cl	C_6_H_11_C_l_N_2_	65039-09-0	0.1466	>0.99	Lanzhou Institute of Chemical Physics, Chinese Academy of Sciences
[Bmim]Cl	C_8_H_15_C_l_N_2_	79917-90-1	0.1747	>0.99	
[Hmim]Cl	C_10_H_19_C_l_N_2_	171058-17-6	0.2027	>0.98	
MEA	NH_2_CH_2_CH_2_OH	141-43-5	0.0611	>0.995	Macklin Inc. (Shanghai, China)
water	H_2_O	7732-18-5	0.0180	ultrapure (5.5 × 10^–6^ S/m)	none

### 2.2 Preparation of DESs

The DESs ([EmimCl]­[MEA] (1:4), [BmimCl]­[MEA] (1:4), and [HmimCl]­[MEA] (1:4)) were prepared by mixing dried ILs with MEA at a molar ratio of 1:4, followed by heating and stirring at 358 K for 2 h. All DESs were prepared gravimetrically using an analytical balance. For instance, 30.0030 g of [Emim]Cl and 49.9917 g of MEA were combined to produce [EmimCl]­[MEA], corresponding to a calculated MEA-to-IL molar ratio of 3.9997 ± 0.0013. Similarly, the MEA-to-IL molar ratios for [BmimCl]­[MEA] and [HmimCl]­[MEA] were determined to be 4.0020 ± 0.0013 and 4.0011 ± 0.0014, respectively. Although the molar ratios were controlled to the third decimal place, all DES compositions are reported as 1:4 throughout this work for clarity and consistency. Accordingly, the molar masses of three prepared DESs are 0.0782, 0.0838, and 0.0894 kg·mol^–1^, respectively. All preparation steps were carried out in a low-humidity glovebox (H_2_O < 0.01 ppm). The resulting [EmimCl]­[MEA] (1:4) and [BmimCl]­[MEA] (1:4) were colorless and transparent liquids, while [HmimCl]­[MEA] (1:4) formed a yellow transparent liquid. All prepared DESs were dehydrated using preactivated 3 Å molecular sieves and subsequently sealed for 2 weeks. No phase separation or degradation was observed during this storage period. The water content of all DESs was measured using a Karl Fischer Moisture Titrator (V100), with a minimum of three replicate measurements per sample. The average water contents were 280 ppm for [EmimCl]­[MEA] (1:4), 340 ppm for [BmimCl]­[MEA] (1:4), and 330 ppm for [HmimCl]­[MEA] (1:4).

The successful formation of the DESs was confirmed through characterization by Fourier-transform infrared spectroscopy (FT-IR), proton nuclear magnetic resonance (^1^H NMR), and carbon-13 nuclear magnetic resonance (^13^C NMR). Detailed spectral data are provided in Figures S1–S5 of the Supporting Information.

Differential scanning calorimetry (DSC, TA Q20 DSC) was employed to determine the melting points (*T*_m_) and glass transition temperatures (*T*_g_) of DESs and their pure components. The DSC measurements were performed over 183–423 K, under a flow rate of 40 mL·min^–1^ of N_2_, using a heating rate of 10 K·min^–1^ and sample masses between 0.01 and 0.15 g. The *T*_m_ of MEA, [Emim]­Cl, [Bmim]­Cl, [EmimCl]­[MEA] (1:4), and [BmimCl]­[MEA] (1:4) were determined to be 281.9, 362.1, 330.7, 265.0, and 267.0 K, respectively. Accounting for the instrumental accuracy of 0.1 K and calibration residuals, the expanded uncertainties (*k* = 2) for the measured phase transition temperatures (*T*_m_ and *T*_g_) were assigned as 0.2 K. The corresponding DSC thermograms are shown in Figure S6. Both [EmimCl]­[MEA] (1:4) and [BmimCl]­[MEA] (1:4) exhibit lower *T*_m_ values than their individual components, confirming the formation of mixtures with typical DES characteristics. For [Hmim]Cl and its corresponding DES, [HmimCl]­[MEA] (1:4), their *T*_m_ values could not be determined because both remained liquid over the entire temperature range investigated. Nevertheless, spectroscopic features of [HmimCl]­[MEA] (1:4) were consistent with those of the DESs derived from [Emim]Cl and [Bmim]­Cl, showing no significant differences in the key vibrational bands with H-bond formation. These observations support the classification of all three systems [EmimCl]­[MEA] (1:4), [BmimCl]­[MEA] (1:4), and [HmimCl]­[MEA] (1:4) as DESs. This classification is further supported by the previous reports of DESs formed from [Bmim]Cl or [Hmim]Cl with trifluoroacetamide, which display comparable thermal behavior.^40^

### 2.3 Measurement of Density and Viscosity

The mixtures of DES and H_2_O were prepared in a vacuum glovebox (<0.1 ppm moisture) using a digital analytical balance (Sartorius SECURA225D-1CN). Prior to the measurement, the samples were tightly sealed and magnetically stirred for over 2 h to ensure uniform mixing.

Density (ρ) measurements were carried out using an oscillating *U*-tube densitometer (Anton Paar, DMA 5000M) equipped with a high-precision thermostat, maintaining a temperature uncertainty at 0.01 K. Prior to each measurement, the instrument was thoroughly cleaned with ultrapure water and anhydrous ethanol, dried using the built-in blower, and calibrated with dry air and ultrapure water. The built-in bubble detection system was employed to ensure bubble-free conditions during measurements. Each measurement was performed at least twice, and the average values were reported.

Viscosity (η) measurements were performed using a rolling-ball viscosimeter (Anton Paar, Lovis 2000 ME) with capillary tubes of various diameters (1.59, 1.80, and 2.50 mm) and a stainless-steel ball bearing with a diameter of 1.5 mm, allowing accurate measurement across a wide viscosity range. The instrument was calibrated using standard viscosity reference liquids (N7.5, N26, and N100). The viscosimeter is equipped with a temperature control system, maintaining a temperature uncertainty of 0.02 K. Each measurement was performed at least twice, and the average value was reported.

### 2.4 Measurement of Enthalpy of Mixing

The enthalpy of mixing (Δ_mix_ *H*) was measured using a TAM isothermal microcalorimeter (TA Instruments, USA) equipped with an air circulation system to ensure stable temperature control, with a fluctuation of ±0.02 K. Instrument accuracy was verified by measured the enthalpy of dissolution of KCl (0.0192 g) in ultrapure water (1.0016 g), yielding −17.50 kJ·mol^–1^, in excellent agreement with the literature value (−17.47 ± 0.07 kJ·mol^–1^).^42^

For each measurement, a known amount of DES was placed in a 20 mL ampule positioned in the measured channel, while the solvent (H_2_O) was loaded into a Hamilton syringe positioned above the ampule. In the reference channel, a corresponding amount of H_2_O, with a comparable heat capacity to the DES sample, was utilized to minimize baseline drift due to external temperature fluctuations. During the experiment, the solvent was automatically injected into the ampule containing the DES, and the resulting mixture was continuously stirred using the built-in mechanical stirrer. The heat flow was continuously monitored, and the thermal response curve was recorded. Once thermal equilibrium was reached, the Δ_mix_ *H* value was calculated by integrating the heat signal. Each measurement was performed at least thrice, and the average value was reported.

### 2.5 Uncertainty Analysis

Using a digital analytical balance with a precision of 1 × 10^–4^ g, the combined expanded uncertainty of mass was estimated to be *U*(*m*) ≈ 5 × 10^–4^ g at a 95% confidence level (coverage factor *k* ≈ 2). Considering the final purity of the reagents (≥0.98), the combined standard uncertainty of mole fraction for each sample was estimated to be *u*(*x*) ≈ 0.002.

For density measurement, the combined expanded uncertainty of density, *U*(ρ), was determined by aggregating the contributors of instrument accuracy, repeatability, and temperature influence. The density meter has an accuracy of 1 × 10^–3^ kg/m^3^, resulting in a synthetic accuracy of 6.5 × 10^–4^ kg/m^3^. The uncertainties derived from repeated measurements and temperature influence were calculated to be 5 × 10^–3^ and 8.7 × 10^–4^ kg/m^3^, respectively. Using the root-sum-square method and a coverage factor of *k* ≈ 2 (representing a 95% confidence level), *U*(ρ) was estimated to be 1 × 10^–2^ kg/m^3^. Furthermore, acknowledging the density measurements are affected by sample purity,^43^ and given that the sample purity exceeds 0.98 (mass fraction), the relative expanded uncertainty of density was estimated as *U*_r_(ρ) ≈ 0.002 (95% level of confidence). Similarly, the viscosity meter has an accuracy of 0.005, yielding a synthetic accuracy of 0.003. The uncertainties derived from repeatability and temperature effect were estimated to be 0.0025 and 0.002, respectively. Therefore, the relative expanded uncertainty of viscosity was estimated to be *U*_r_(η) ≈ 0.01 at the 95% confidence level. Uncertainties associated with the Δ_mix_ *H* at each mole fraction were discussed in the subsequent section.

## 3 Results and Discussion

### 3.1 Comparison of Measurement

The densities and viscosities of ([EmimCl]­[MEA] (1:4) + H_2_O), ([BmimCl]­[MEA] (1:4) + H_2_O), and ([HmimCl]­[MEA] (1:4) + H_2_O) systems were measured across a temperature range of 288.15–323.15 K at ambient pressure, with the measured results summarizing in Tables 2 and 3.

**Table 2:** Densities (ρ) of ([EmimCl]­[MEA] (1:4) + H_2_O), ([BmimCl]­[MEA] (1:4) + H_2_O) and ([HmimCl]­[MEA] (1:4) + H_2_O) at Different Temperatures (*T*) and Mole Fractions (*x*_1_) at 1.01 × 10^5^ Pa^a^

	*T*, K							
*x*_1_	288.15	293.15	298.15	303.15	308.15	313.15	318.15	323.15
ρ (kg·m^–3^) – [EmimCl][MEA] (1:4) (1) + H_2_O (2)								
0.000	999.22	998.33	997.18	995.79	994.18	992.37	990.38	988.21
0.100	1028.14	1026.05	1023.86	1021.54	1019.12	1016.59	1013.95	1011.21
0.200	1048.91	1046.09	1043.21	1040.27	1037.27	1034.22	1031.10	1027.93
0.301	1061.52	1058.33	1055.11	1051.86	1048.57	1045.24	1041.88	1038.48
0.399	1068.42	1065.06	1061.69	1058.29	1054.87	1051.42	1047.95	1044.45
0.495	1071.23	1067.78	1064.33	1060.85	1057.36	1053.85	1050.32	1046.77
0.596	1072.21	1068.69	1065.17	1061.64	1058.10	1054.56	1051.00	1047.43
0.703	1071.33	1067.76	1064.19	1060.62	1057.04	1053.46	1049.88	1046.29
0.796	1070.87	1067.26	1063.68	1060.08	1056.48	1052.88	1049.28	1045.69
0.901	1069.24	1065.60	1061.98	1058.35	1054.74	1051.13	1047.53	1043.94
1.000	1068.08	1064.41	1060.77	1057.14	1053.51	1049.90	1046.29	1042.70
ρ (kg·m^–3^) – [BmimCl][MEA] (1:4) (1) + H_2_O (2)								
0.000	999.22	998.33	997.18	995.79	994.18	992.37	990.38	988.21
0.100	1025.41	1023.13	1020.74	1018.24	1015.64	1012.94	1010.14	1007.25
0.201	1042.69	1039.64	1036.54	1033.40	1030.20	1026.96	1023.66	1020.31
0.300	1050.77	1047.44	1044.09	1040.71	1037.29	1033.85	1030.36	1026.85
0.382	1053.64	1050.22	1046.79	1043.33	1039.85	1036.34	1032.80	1029.24
0.498	1054.39	1050.91	1047.43	1043.93	1040.41	1036.87	1033.32	1029.75
0.597	1053.02	1049.51	1045.99	1042.46	1038.92	1035.37	1031.81	1028.24
0.703	1051.53	1047.99	1044.44	1040.89	1037.34	1033.78	1030.22	1026.65
0.802	1050.09	1046.52	1042.95	1039.39	1035.83	1032.27	1028.71	1025.15
0.900	1048.43	1044.84	1041.27	1037.69	1034.13	1030.56	1027.01	1023.46
1.000	1046.89	1043.26	1039.64	1036.03	1032.43	1028.85	1025.30	1021.76
ρ (kg·m^–3^) – [HmimCl][MEA] (1:4) (1) + H_2_O (2)								
0.000	999.22	998.33	997.18	995.79	994.18	992.37	990.38	988.21
0.100	1035.93	1033.48	1030.93	1028.28	1025.52	1022.68	1019.73	1016.69
0.202	1057.22	1054.07	1050.86	1047.61	1044.31	1040.95	1037.54	1034.07
0.303	1068.40	1064.98	1061.53	1058.04	1054.52	1050.97	1047.37	1043.73
0.402	1074.05	1070.52	1066.96	1063.38	1059.76	1056.12	1052.46	1048.76
0.496	1076.72	1073.13	1069.52	1065.89	1062.24	1058.56	1054.86	1051.15
0.602	1077.84	1074.20	1070.55	1066.88	1063.20	1059.51	1055.80	1052.08
0.700	1078.05	1074.36	1070.68	1066.99	1063.30	1059.59	1055.88	1052.16
0.804	1077.77	1074.06	1070.36	1066.66	1062.94	1059.23	1055.52	1051.81
0.899	1077.04	1073.31	1069.61	1065.89	1062.18	1058.46	1054.75	1051.04
1.000	1076.25	1072.50	1068.76	1065.03	1061.30	1057.59	1053.88	1050.17

a Standard uncertainties are *u*(*x*_1_) = 0.002, *u*(*T*) = 0.01 K, *u*(*p*) = 3 × 10^3^ Pa. The relative expanded uncertainty is *U*_r_(ρ) ≈ 0.002 (0.95 level of confidence).

**Table 3:** Viscosities (η) of ([EmimCl]­[MEA] (1:4) + H_2_O), ([BmimCl]­[MEA] (1:4) + H_2_O), and ([HmimCl]­[MEA] (1:4) + H_2_O) at Different Temperatures (*T*) and Mole Fractions (*x*_1_) at 1.01 × 10^5^ Pa^a^

	*T*, K							
*x*_1_	288.15	293.15	298.15	303.15	308.15	313.15	318.15	323.15
η, 10^–3^ Pa·s – [EmimCl][MEA] (1:4) (1) + H_2_O (2)								
0.000	1.14	1.00	0.90	0.80	0.73	0.67	0.61	0.57
0.100	3.22	2.76	2.39	2.08	1.83	1.62	1.44	1.29
0.200	7.44	6.12	5.11	4.31	3.70	3.19	2.78	2.44
0.301	13.62	10.96	8.95	7.40	6.18	5.23	4.47	3.90
0.399	20.35	16.10	12.94	10.57	8.75	7.33	6.20	5.30
0.495	26.55	20.55	16.27	13.14	10.79	8.96	7.54	6.39
0.596	30.63	23.85	18.93	15.26	12.47	10.33	8.65	7.33
0.703	33.98	26.49	20.94	16.79	13.69	11.34	9.51	8.08
0.796	36.19	27.92	22.08	17.73	14.45	12.00	10.08	8.56
0.901	36.47	28.28	22.29	17.85	14.58	12.09	10.16	8.64
1.000	36.77	28.64	22.52	18.09	14.76	12.25	10.34	8.83
η, 10^–3^ Pa·s – [BmimCl][MEA] (1:4) (1) + H_2_O (2)								
0.000	1.14	1.00	0.90	0.80	0.73	0.67	0.61	0.57
0.100	3.65	3.09	2.64	2.29	2.00	1.76	1.56	1.39
0.201	8.74	7.11	5.88	4.92	4.19	3.60	3.11	2.71
0.300	16.10	12.77	10.30	8.45	7.02	5.89	5.00	4.29
0.382	23.43	18.36	14.62	11.81	9.56	7.95	6.67	5.66
0.498	32.12	24.90	19.96	15.98	12.87	10.60	8.88	7.51
0.597	37.23	28.61	22.34	17.78	14.38	11.78	9.79	8.23
0.703	41.81	32.11	25.01	20.02	16.08	13.16	10.96	9.21
0.802	44.19	33.83	26.53	21.20	17.12	14.02	11.65	9.81
0.900	47.23	36.25	28.25	22.42	18.15	14.91	12.40	10.44
1.000	47.77	36.41	28.51	22.64	18.25	14.99	12.50	10.54
η, 10^–3^ Pa·s – [HmimCl][MEA] (1:4) (1) + H_2_O (2)								
0.000	1.14	1.00	0.90	0.80	0.73	0.67	0.61	0.57
0.100	3.77	3.17	2.70	2.33	2.03	1.79	1.58	1.41
0.202	9.05	7.36	6.07	5.08	4.30	3.71	3.21	2.80
0.303	16.97	13.46	10.84	8.85	7.32	6.12	5.19	4.44
0.402	25.76	20.13	15.94	12.83	10.49	8.68	7.29	6.17
0.496	34.45	26.93	21.03	16.69	13.46	11.03	9.16	7.71
0.602	40.71	31.10	24.20	19.17	15.43	12.66	10.49	8.82
0.700	45.72	34.96	27.22	21.40	17.41	14.13	11.67	9.78
0.804	49.67	38.24	29.77	23.52	18.89	15.40	12.73	10.66
0.899	52.44	40.29	31.24	24.67	19.86	16.21	13.46	11.30
1.000	54.33	40.82	31.68	25.07	20.18	16.48	13.66	11.46

a Standard uncertainties are *u*(*x*_1_) = 0.002, *u*(*T*) = 0.01 K, *u*(*p*) = 3 × 10^3^ Pa. The relatively expanded uncertainty is *U*_r_(η) ≈ 0.01 (0.95 level of confidence).

To validate the experimental densities and viscosities, our measurements for [BmimCl]­[MEA] (1:4),^27^ H_2_O,^44–51^ and MEA^52–57^ were compared against available literature data. The density and viscosity data obtained in this work for MEA are provided in Table S1. The agreement was quantified using the relative deviation (δ), calculated as δ = 100­(*Y*_exp_ – *Y*_fit_)/*Y*_fit_ (where *Y* represents either ρ or η). The fitted density and viscosity data for [BmimCl]­[MEA] (1:4), MEA, and H_2_O are summarized in Tables S2 and S3. A visual comparison of the experimental and literature values, including the corresponding relative deviations, is presented in Figures 1 and 2.

**Figure 1:**
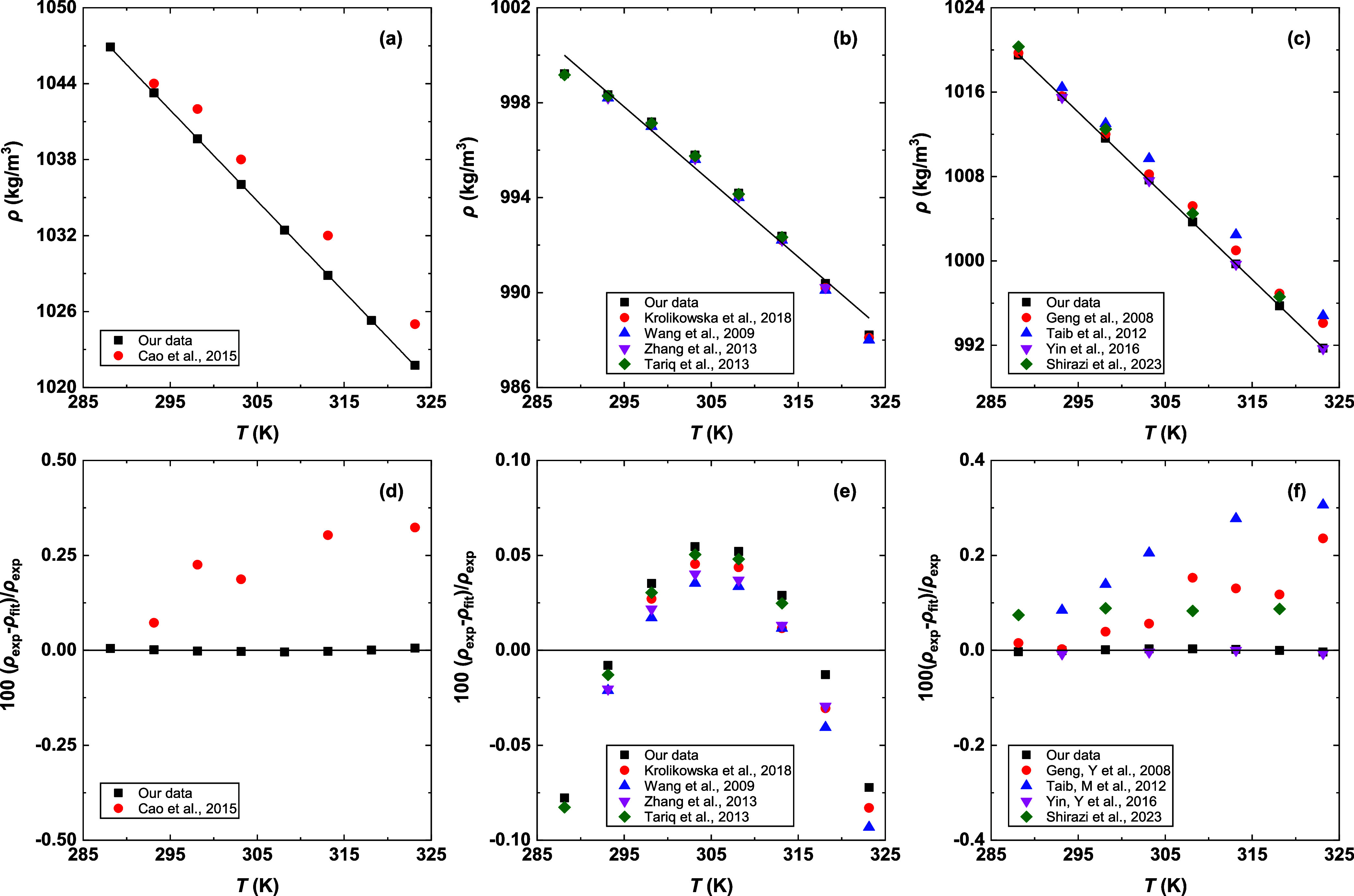
Comparison of experimental density and available literature data for (a) [BmimCl]­[MEA] (1:4),^27^ (b) H_2_O,^44–47^ (c) MEA,^52,53,55–57^ and (e,f) their corresponding deviations. Solid lines in (a–c) indicate the fitted results, and the black solid lines in (e,f) indicate the zero line.

**Figure 2:**
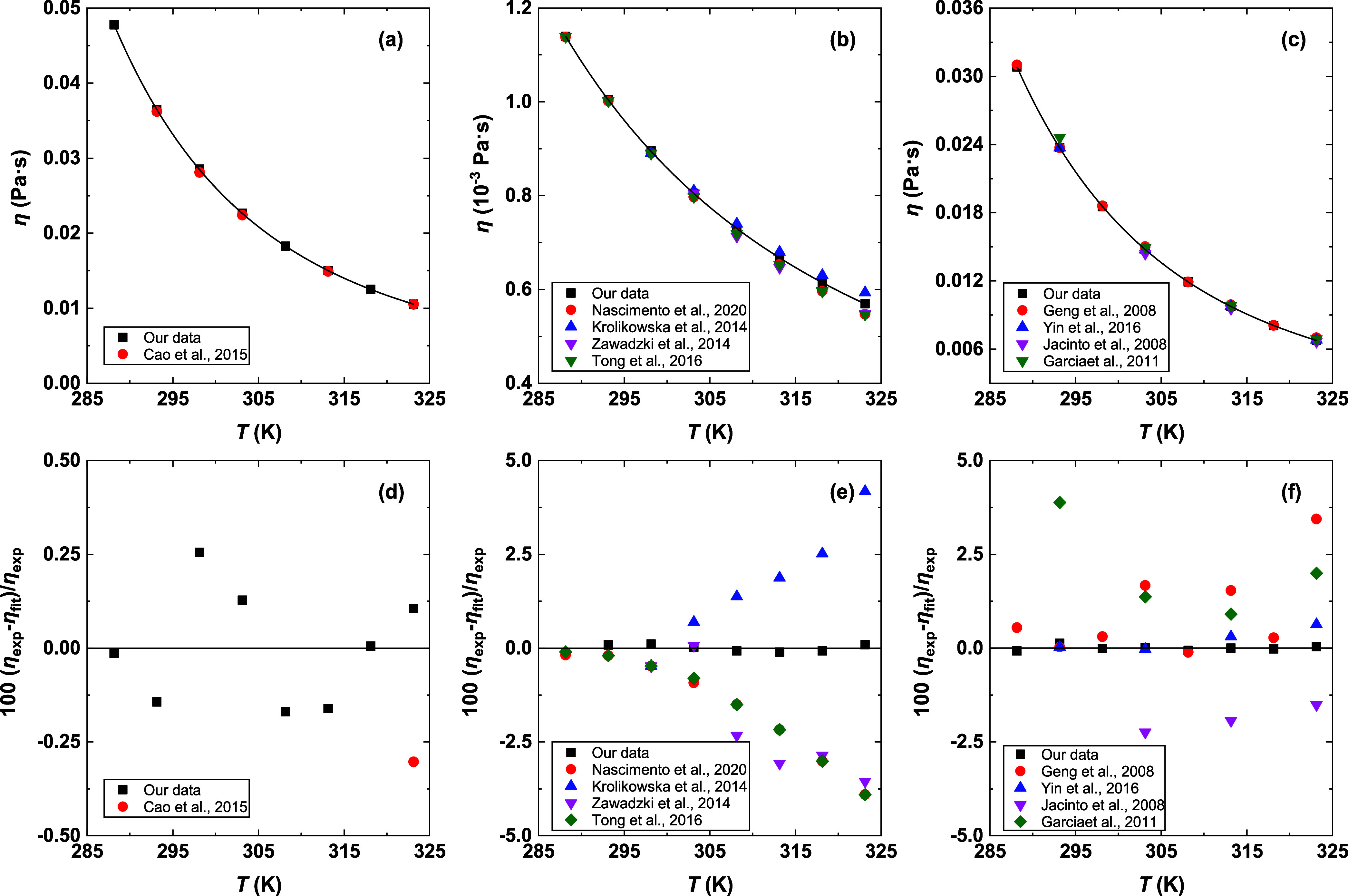
Comparison of experimental viscosity and available literature data for (a) [BmimCl]­[MEA] (1:4),^27^ (b) H_2_O,^48–51^ (c) MEA,^52–54,56^ and (e,f) their corresponding deviations. Solid lines in (a–c) indicate the fitted results, and the black solid lines in (e,f) indicate the zero line.

For MEA and H_2_O, the average relative deviations (ARDs) for density are found to be below 0.2% (Figure 1), which is consistent with our estimated experimental uncertainty. For [BmimCl]­[MEA] (1:4), the density reported by Cao et al.^27^ exhibited a difference of 0.25% from our measured results. While this value slightly exceeds the relative expanded uncertainty, the overall comparisons confirm the accuracy of our density measurements. The measured viscosities for [BmimCl]­[MEA] (1:4) show good agreement with the literature data, characterized by ARDs lower than 1.0% (Figure 2). For MEA and H_2_O, the measured viscosities are generally consistent with the reported literature values, with the present data falling within the range of literature results. Therefore, these comparisons confirm the accuracy of both our density and viscosity measurements.

### 3.2 Density and Viscosity of DES–H_2_O Mixtures

According to the experimental data presented in Tables 2 and 3, the temperature- and composition-dependent trends in density and viscosity are illustrated in Figures 3 and 4, respectively. Figure 3a shows a linear decrease in density with increasing temperature for all studied mixtures. The density differences between compositions are more pronounced at mole fractions between 0.1 and 0.5 than between 0.5 and 0.9. This observation indicates the density is more sensitive to compositional changes in the water-rich region, suggesting more substantial changes in intermolecular interactions and structures within this composition range. Figure 3b presents the variations of density with DES composition and reveals a nonmonotonic dependence on composition. Specifically, the density of the DES–H_2_O mixtures initially increases with the addition of water, reaching a maximum near *x*_DES_ ≈ 0.4–0.5, then decreases with further water addition. This behavior differs from the monotonic density trends typically reported for choline chloride-based DES systems.^5,16^ The observed maximum demonstrates that the initial addition of water molecules enhances molecular packing by penetrating the DES H-bond network, whereas excess water disrupts this network, ultimately leading to the expansion of the liquid structure. At a fixed temperature, the densities of the pure DESs follow the order [HmimCl]­[MEA] (1:4) > [EmimCl]­[MEA] (1:4) > [BmimCl]­[MEA] (1:4). This sequence deviates from the typical trend of decreasing density with increasing alkyl-chain length. The relatively high density of [HmimCl]­[MEA] (1:4) likely arises from more efficient molecular packing, possibly facilitated by stronger H-bond interactions between [Hmim]^+^ and MEA, which compensate for the volumetric expansion typically associated with the longer alkyl substituent.

**Figure 3:**
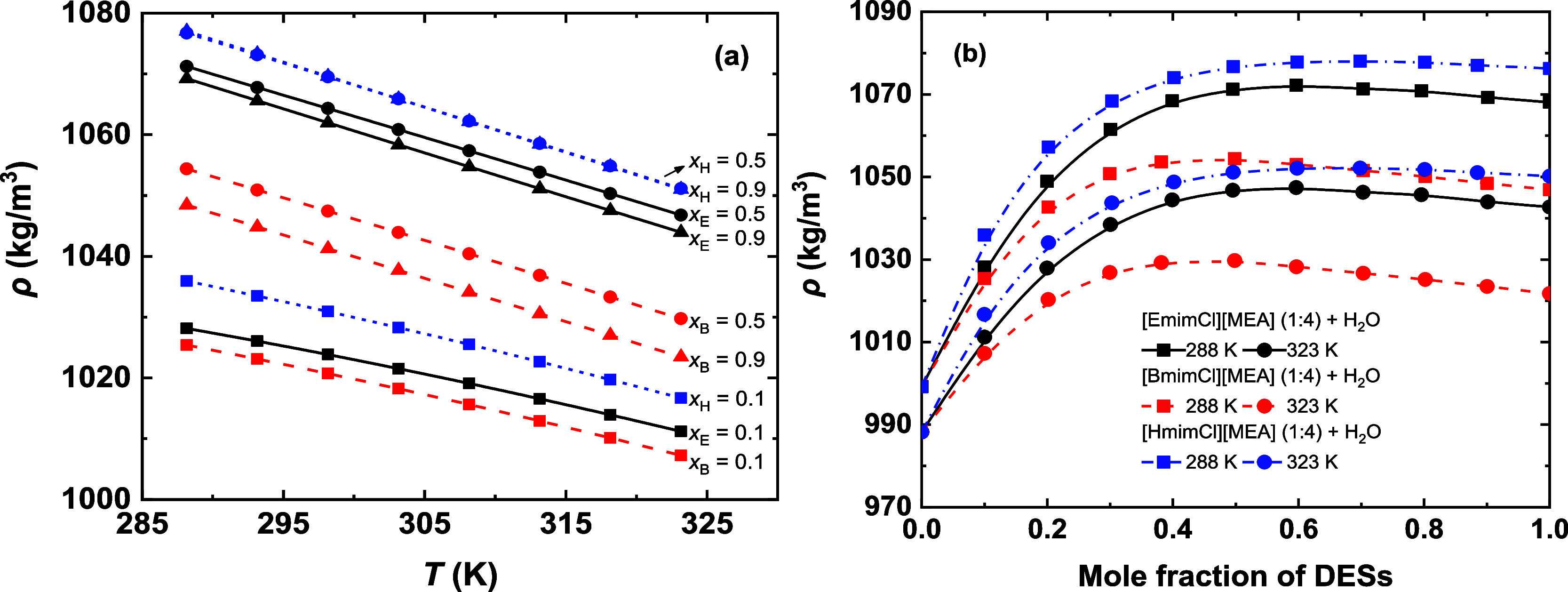
Density variations of the studied systems as a function of (a) temperature and (b) DES concentration. The variables *x*_E_, *x*_B_, and *x*_H_ represent the mole fractions of [EmimCl]­[MEA] (1:4), [BmimCl]­[MEA] (1:4), and [HmimCl]­[MEA] (1:4), respectively, in the corresponding DES–H_2_O systems. Curves are provided as visual guides.

**Figure 4:**
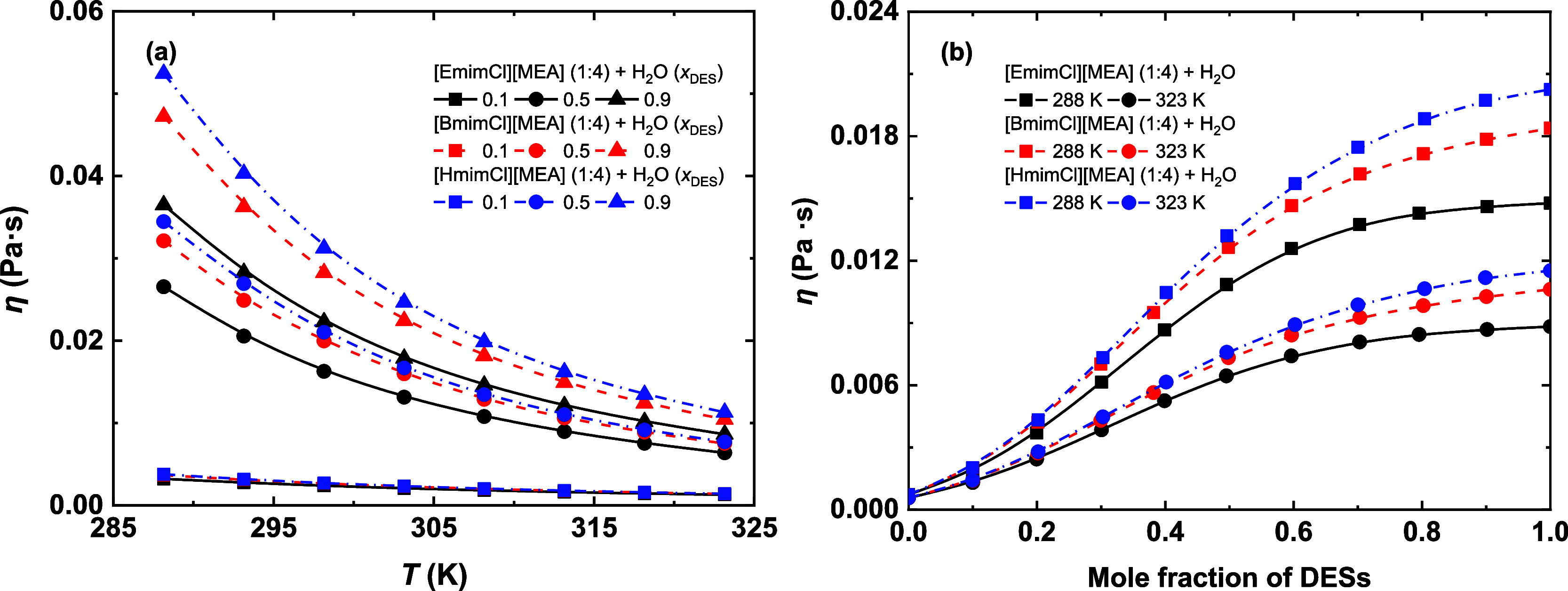
Viscosity of [EmimCl]­[MEA] (1:4), [BmimCl]­[MEA] (1:4), and [HmimCl]­[MEA] (1:4) as a function of (a) temperature and (b) DES concentration. Curves are provided as visual guides.

Figure 4a presents the temperature dependence of viscosity for the studied systems at representative DES contents (*x*_DES_ = 0.1, 0.5, and 0.9). In all cases, viscosity decreases exponentially with increasing temperature, consistent with Arrhenius-type behavior. The viscosity of DES-rich mixtures exhibits greater temperature sensitivity than that of water-rich mixtures, indicating higher activation energies for viscous flow in DES-rich mixtures. Figure 4b displays the composition dependence of viscosity at 288.15 and 323.15 K. Viscosity increases sharply with increasing the DES content up to *x*_DES_ ≈ 0.6 and then rises more gradually as the mixture approaches pure DES. This behavior may be attributed to significant disruptions of H-bond networks in the H_2_O-rich region, whereas those in the DES-rich region remain relatively stable. Among the studied systems, the ([HmimCl]­[MEA] (1:4) + H_2_O) exhibits the highest viscosity, while the ([EmimCl]­[MEA] (1:4) + H_2_O) showed the lowest, correlating with the increasing alkyl chain length of the imidazolium cation.

### 3.3 Excess Molar Volumes and Viscosity Deviation

The nonideal behavior of the studied systems, which results from structural rearrangement or specific molecular interactions, is quantified using the excess molar volumes (*V* ^E^) and the viscosity deviations (Δη). These excess properties were calculated using the following equation

1 $$V^{E}=\frac{x_{1}M_{1}+x_{2}M_{2}}{\rho }-\frac{x_{1}M_{1}}{\rho_{1}^{*}}-\frac{x_{2}M_{2}}{\rho_{2}^{*}}$$

2 $$\Delta \eta =\eta -x_{1}\eta_{1}-x_{2}\eta_{2}$$

where *x*_*i*_, *M*_*i*_, ρ_i_^*^, and η_*i*_ are the mole fraction, molar weight, density, and viscosity of component *i*, respectively. ρ and η are the density and viscosity of binary mixture, respectively. The calculated *V* ^E^ and Δη values, which quantify the nonideal mixing behavior, are presented in Tables S4 and S5, respectively.

The relationship between *V* ^E^ or Δη and mixture composition was correlated using the Redlich–Kister (RK) polynomial equation

3 $$V^{E}\left(\Delta \eta \right)=x_{1}x_{2}\sum_{j=0}^{k}A_{j}{\left(x_{1}-x_{2}\right)}^{j}$$

where *A*_*j*_ are the RK polynomial coefficients, and *k* is the polynomial order. In this work, we set *k* = 4, and the corresponding *A*_*j*_ parameters are listed in Tables S6 and S7, respectively.

Figure 5 illustrates the *V* ^E^ of the ([EmimCl]­[MEA] (1:4) + H_2_O), ([BmimCl]­[MEA] (1:4) + H_2_O), and ([HmimCl]­[MEA] (1:4) + H_2_O) mixtures as functions of composition at different temperatures. The *V* ^E^ values are negative across the entire concentration range, indicating volume contraction upon the mixing. The magnitude of |*V* ^E^| exhibits pronounced minima near *x*_DES_ ≈ 0.4–0.5 (corresponding to a water mass fraction of about 0.2), coinciding with the turning points observed in the density–composition curves. This correlation confirms the formation of the most compact liquid structures at such concentrations. With increasing temperature, |*V* ^E^| decreases for all systems, reflecting the progressive weakening of H-bond interactions and allowing the system to approach ideal mixing behavior. This trend suggests that H-bond interactions are the dominant factor contributing to the observed deviations from ideality. A comparative analysis of *V* ^E^ among the three systems reveals that the alkyl chain length of the imidazolium cation exerts only a minor influence on the magnitude of *V* ^E^. Overall, the degree of nonideality follows the order [HmimCl]­[MEA] (1:4) > [EmimCl]­[MEA] (1:4) > [BmimCl]­[MEA] (1:4), which is consistent with the order observed in density results.

**Figure 5:**
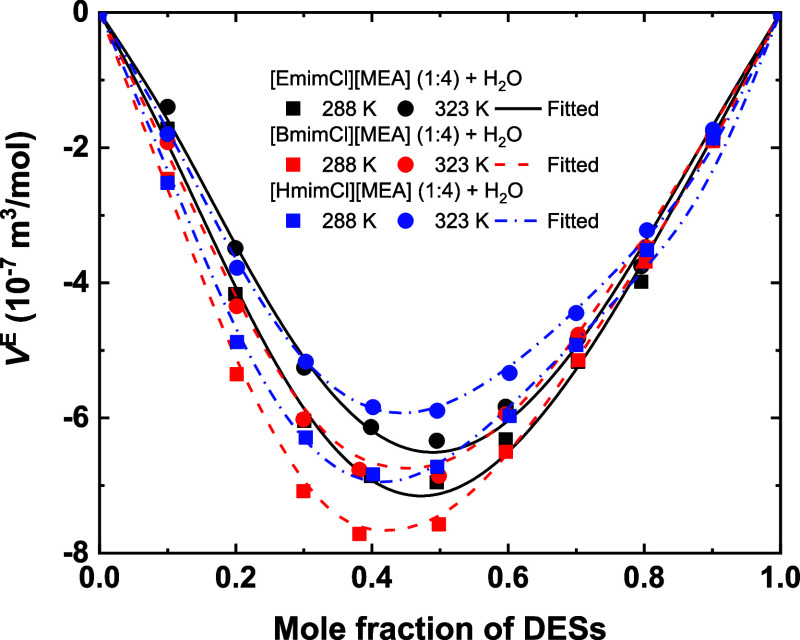
Excess volumes of the studied systems as a function of composition at 288.15 and 323.15 K. Symbols represent experimental data; curves represent fitted results of the RK polynomial expression.

Figure 6 illustrates Δη for the same systems at 288.15 and 323.15 K. Δη exhibits an “S-shaped” dependence on composition: negative in the water-rich region and positive in the DES-rich region. Negative Δη arises from H-bond network disruption and enhanced mobility at low DES content, while positive Δη reflects H-bond network formation and strong associative interactions at higher DES concentrations. With increasing temperature, |Δη| decreases for all systems, consistent with weakened H-bond interactions. The extent of deviation at low DES content follows the order [HmimCl]­[MEA] (1:4) > [BmimCl]­[MEA] (1:4) > [EmimCl]­[MEA] (1:4), whereas at high DES content, the trend is generally reversed.

**Figure 6:**
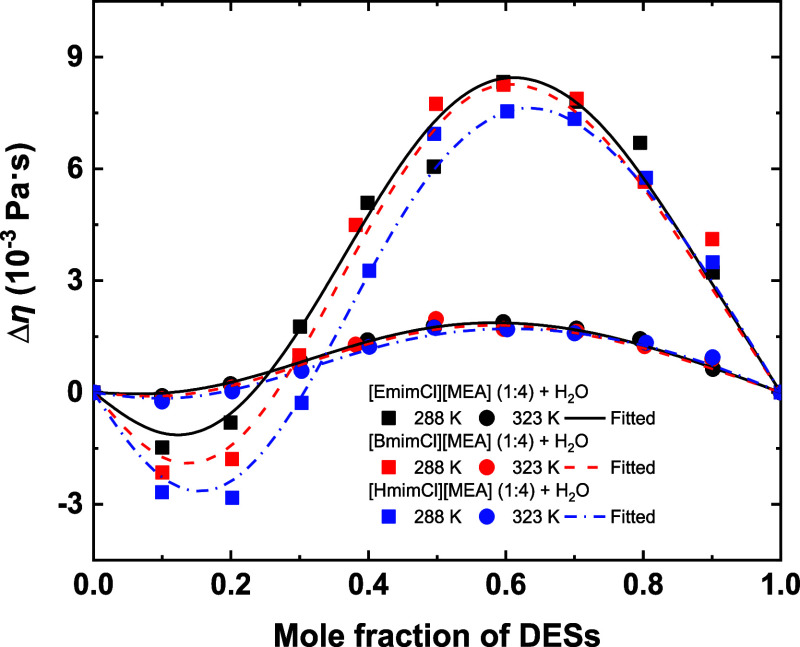
Viscosity deviations of the studied systems as a function of composition at 288.15 and 323.15 K. Symbols represent experimental data; curves represent fitted results of the RK polynomial expression.

### 3.4 Enthalpy of Mixing and Thermodynamic Modeling

#### 3.4.1 Experimental Data

The enthalpies of mixing (Δ_mix_ *H*) for the ([EmimCl]­[MEA] (1:4) + H_2_O), ([BmimCl]­[MEA] (1:4) + H_2_O), and ([HmimCl]­[MEA] (1:4) + H_2_O) systems were measured over the full composition range at 298.15 and 308.15 K. The experimental values and associated uncertainties are summarized in Table S8. The concentration-dependent variations of Δ_mix_ *H* at both temperatures are depicted in Figure 7. All measured Δ_mix_ *H* values are negative, indicating exothermic and energetically favorable mixing driven by strong specific interactions, primarily H-bond interactions, between DES components and water. Each system exhibits a distinct minimum in Δ_mix_ *H* near *x*_DES_ ≈ 0.4–0.5, matching the composition of the *V* ^E^ minima and the viscosity transition region. These features suggest a reorganization of the H-bond network and the possible formation of ternary, DES-like associated structures, leading to enhanced molecular packing and increased resistance to flow. In other words, at intermediate water contents, DES-like structures may be largely preserved while the viscosity is significantly reduced, which is particularly relevant for DES-specific applications such as CO_2_ capture and extraction processes.^58,59^ Among the studied systems, ([HmimCl]­[MEA] (1:4) + H_2_O) displays the least exothermic mixing behavior, followed by ([BmimCl]­[MEA] (1:4) + H_2_O) and ([EmimCl]­[MEA] (1:4) + H_2_O). Based on the density and viscosity results, it is inferred that [HmimCl]­[MEA] (1:4) forms stronger interactions, and its weaker exothermicity likely arises from the greater energy required to disrupt the H-bond network within the mixture.

**Figure 7:**
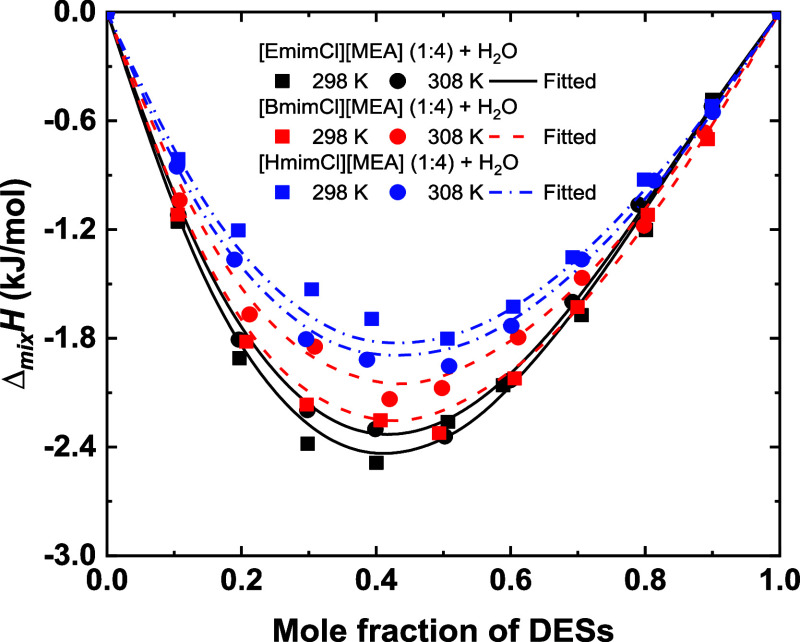
Enthalpies of mixing of the studied systems as a function of composition at 298.15 and 308.15 K. Symbols represent experimental data; curves represent fitted results of the NRTL model.

#### 3.4.2 Modeling Based on NRTL

The relationship between Δ_mix_ *H* and excess Gibbs free energy (*G* ^E^) can be described using the Gibbs–Helmholtz equation, which is given as

4 $$\Delta_{mix}H=-RT^{2}{\left[\frac{\partial \left(G^{E}/RT\right)}{\partial T}\right]}_{p,x}$$

where *R* is the gas constant. *G* ^E^ can be calculated by NRTL equation, which has been widely employed to correlate thermodynamic properties of binary liquid mixtures, such as vapor–liquid equilibrium (VLE) and enthalpy of mixing. We aim to evaluate the suitability of the NRTL model for correlating Δ_mix_ *H* data of systems containing DESs and H_2_O. The determined interaction parameters can subsequently be combined with VLE calculations to estimate the vapor pressure of H_2_O in DES-based systems, thereby providing essential thermodynamic input for the design and optimization of chemical processes, such as CO_2_ capture. For a binary mixture, *G* ^E^ is expressed as

5 $$G^{E}=x_{1}x_{2}\left[\frac{\tau_{21}G_{21}}{x_{1}+x_{2}G_{21}}+\frac{\tau_{12}G_{12}}{x_{2}+x_{1}G_{12}}\right]$$

where τ_12_ = Δ*g*_11_ + Δ*g*_12_/*T*, τ_21_ = Δ*g*_21_ + Δ*g*_22_/*T*, *G*_12_ = exp­(−α_12_τ_12_), and *G*_21_ = exp­(−α_21_τ_21_). Combining eqs 4 and 5 can derive the expression of Δ_mix_ *H* from the NRTL model, which is given by

6 $$\Delta_{mix}H=-Rx_{1}x_{2}\left\{\frac{\Delta g_{21}G_{21}\left[x_{1}\left(\alpha_{21}\tau_{21}-1\right)-x_{2}G_{21}\right]}{{\left(x_{1}+x_{2}G_{21}\right)}^{2}}+\frac{\Delta g_{12}G_{12}\left[x_{2}\left(\alpha_{12}\tau_{12}-1\right)-x_{1}G_{12}\right]}{{\left(x_{2}+x_{1}G_{12}\right)}^{2}}\right\}$$

In these equations, Δ*g*_11_, Δ*g*_12_, Δ*g*_21_, and Δ*g*_22_ serve as adjustable fitting parameters, whereas α_12_ and α_21_ denote the nonrandomness factors. For DES systems, α is typically set to 0.2,^60^ and the same value was adopted in this work. The resulting parameters are compiled in Table S9 together with their associated ARDs. A comparison of the experimental results with the model predictions is shown in Figure 7, indicating good alignment across the three studied systems, with ARDs remaining below 5.0%.

### 3.5 Further Comparison with Subsystems

The comparison of the physicochemical properties of DES–H_2_O systems with those of their individual components, namely HBA, HBD, and H_2_O, is essential for elucidating their structural and interaction behavior upon water addition. Among the HBAs considered, [Hmim]Cl was selected for further analysis due to its extensive experimental data and its ability to remain in the liquid state across the entire concentration range, in contrast to [Emim]Cl and [Bmim]­Cl. Experimental data for the densities, viscosities, and enthalpies of mixing of the ([Hmim]Cl + H_2_O)^33,34,61^ and (MEA + H_2_O)^62,63^ systems were collected from the literature and compared with those obtained for the ([HmimCl]­[MEA] (1:4) + H_2_O) system under comparable conditions.

As shown in Figure 8a,b, the ([HmimCl]­[MEA] (1:4) + H_2_O) system exhibits the highest ρ and the most negative *V* ^E^ among the studied systems. This behavior suggests the formation of a greater number of complex intermolecular associations, such as a mixed H-bond network involving ions, H_2_O, and MEA molecules, compared to the MEA–H_2_O or IL–H_2_O clusters present in the other systems.

**Figure 8:**
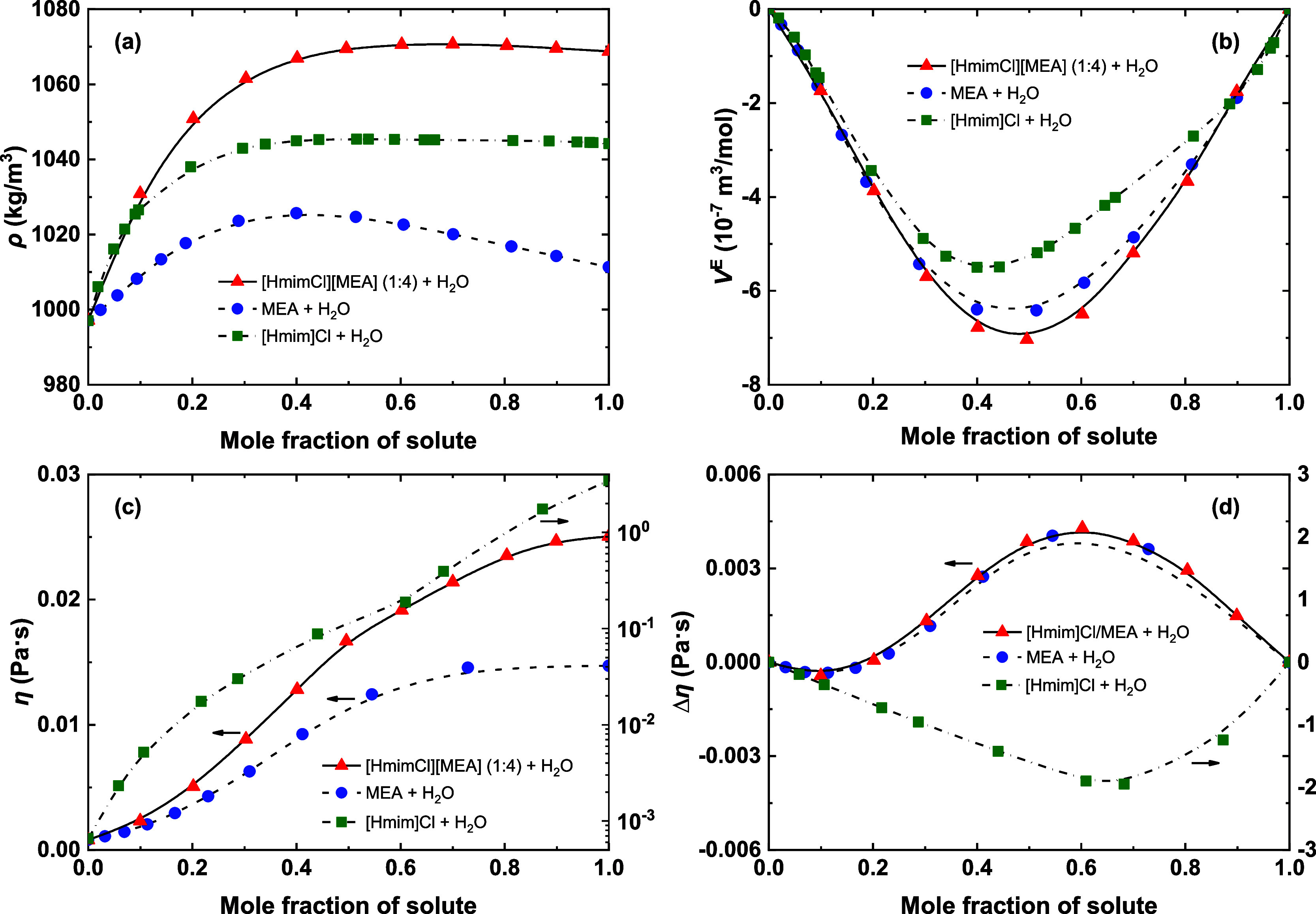
Variations of (a) density, (b) excess molar volumes, (c) viscosity, and (d) viscosity deviation as a function of solute mole fraction for ([HmimCl]­[MEA] (1:4) + H_2_O), (MEA + H_2_O), and ([Hmim]Cl + H_2_O) systems at 298.15 K.

Interestingly, the trends in η and Δη differ from those of ρ. The variations in η and Δη for ([HmimCl]­[MEA] (1:4) + H_2_O) closely resemble those for the (MEA + H_2_O) mixture, both displaying similar η–*x* profiles and S-shaped curves in Δη (Figure 8c,d). In contrast, the ([Hmim]Cl + H_2_O) system shows different behavior, displaying the strongest nonidealities among the three systems, which suggests that water addition significantly disrupts the original IL structures. Because Δη is highly sensitive to the disruption and reorganization of H-bond networks, these results imply that H-bond in the ([HmimCl]­[MEA] (1:4) + H_2_O) mixture primarily occurs between MEA and H_2_O molecules rather than between the ionic species and MEA or H_2_O individually. Combined with the density results, this suggests that water incorporation induces a reorganization of the H-bond networks of [HmimCl]­[MEA] (1:4), leading to the formation of stable MEA–H_2_O H-bond networks that may be interconnected with the HBA cation and anion.

As illustrated in Figure 9, the absolute values of Δ_mix_ *H* follow the order ([Hmim]Cl + H_2_O) > (MEA + H_2_O) > ([HmimCl]­[MEA] (1:4) + H_2_O). This sequence indicates that H_2_O addition to [Hmim]Cl causes a pronounced disruption of its ionic structure and the formation of strong ion–H_2_O interactions, resulting in large exothermic effects. In contrast, the incorporation of H_2_O into [HmimCl]­[MEA] (1:4) also promotes the formation of new interactions, but to a lesser extent than in the ([Hmim]Cl + H_2_O) system. The relatively smaller |Δ_mix_ *H*| observed for ([HmimCl]­[MEA] (1:4) + H_2_O) likely arises from a reorganization of the pre-existing H-bond network. This structural reorganization leads to a more compact and well-ordered liquid structure. The (MEA + H_2_O) system exhibits a similar trend to ([HmimCl]­[MEA] (1:4) + H_2_O, reflecting comparable H-bond rearrangements upon H_2_O addition.

**Figure 9:**
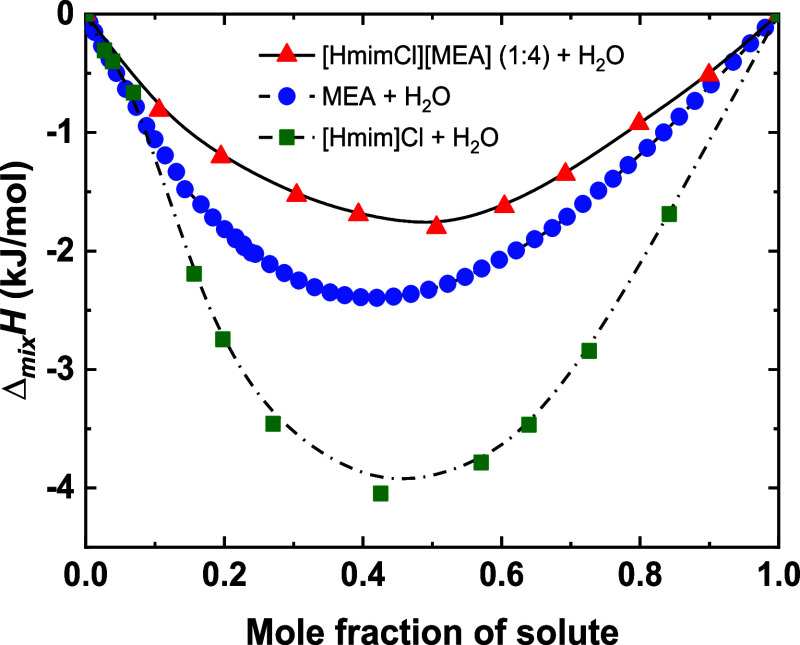
Variations of enthalpy of mixing as a function of solute mole fraction for ([HmimCl]­[MEA] (1:4) + H_2_O), (MEA + H_2_O), and ([Hmim]Cl + H_2_O) systems at 298.15 K.

## 4 Conclusions

The densities and viscosities of the ([C_n_mimCl]­[MEA] + H_2_O) systems (*n* = 2, 4, 6) were systematically measured over 288.15–323.15 K, and the enthalpies of mixing (Δ_mix_ *H*) were determined at 298.15 and 308.15 K. The density data show nonmonotonic composition dependence with extrema at *x*_DES_ ≈ 0.4–0.5, while negative excess molar volumes indicate volume contraction and enhanced molecular packing upon mixing. Viscosity increases sharply at low DES contents and then more gradually toward the pure DESs. The resulting S-shaped viscosity deviation profiles suggest pronounced structural arrangements in the DES-rich and H_2_O-rich composition regions. Furthermore, negative Δ_mix_ *H* values confirm exothermic mixing and strong DES-water interactions, and the experimental data are well correlated by the NRTL model.

Although macroscopic properties alone cannot unambiguously distinguish dilution effects from DES–water structure formation, the consistent trends observed across multiple properties, together with comparative analysis of the ([HmimCl]­[MEA] (1:4) + H_2_O) systems with their subsystems, provide indirect evidence beyond a simple dilution effect. In particular, the coincidence of volume contraction, exothermic mixing, and a pronounced viscosity transition within a narrow compositional range indicates that water does not behave as an inert diluent. Instead, water addition induces reorganization of the H-bond networks, likely involving associated species among ions, H_2_O, and MEA, as well as the formation of an extended H-bond network dominated by MEA–water interactions. Molecular-level insights would require spectroscopic or simulation studies, which are beyond the scope of the present work and will be pursued in further studies.

## Supplementary Material



## Notes

[^1^H NMR, ^13^C NMR, FTIR, and DSC for the studied chemicals; Density and viscosity of MEA at different temperatures; Fitted parameters for variation of density and viscosity with temperatures for H_2_O, MEA, and [BmimCl]­[MEA] (1:4); Excess molar volumes and viscosity deviations for binary mixtures, along with corresponding parameters fitted by the RK equation; Enthalpy of mixing for binary mixtures, along with corresponding parameters fitted by the NRTL model] (PDF)

## References

[1] Metz, B. ; Davidson, O. ; De Coninck, H. ; Loos, M. ; Meyer, L. IPCC Special Report on Carbon Dioxide Capture and Storage; Cambridge University Press: Cambridge, 2005.

[2] Meng F., Meng Y., Ju T., Han S., Lin L., Jiang J. (2022). Research Progress of Aqueous Amine Solution for CO_2_ Capture:
A Review. Renewable Sustainable Energy Rev..

[3] García G., Aparicio S., Ullah R., Atilhan M. (2015). Deep Eutectic
Solvents:
Physicochemical Properties and Gas Separation Applications. Energy Fuels.

[4] Smith E. L., Abbott A. P., Ryder K. S. (2014). Deep Eutectic Solvents (DESs) and
Their Applications. Chem. Rev..

[5] Ma C., Laaksonen A., Liu C., Lu X., Ji X. (2018). The peculiar
effect of water on ionic liquids and deep eutectic solvents. Chem. Soc. Rev..

[6] Hansen B. B., Spittle S., Chen B., Poe D., Zhang Y., Klein J. M., Horton A., Adhikari L., Zelovich T., Doherty B. W., Gurkan B., Maginn E. J., Ragauskas A., Dadmun M., Zawodzinski T. A., Baker G. A., Tuckerman M. E., Savinell R. F., Sangoro J. R. (2021). Deep Eutectic Solvents: A Review
of Fundamentals and Applications. Chem. Rev..

[7] Zhang Y., Ji X., Lu X. (2018). Choline-based
deep eutectic solvents for CO_2_ separation: Review and thermodynamic
analysis. Renewable Sustainable Energy Rev..

[8] Li Z., Wang L., Li C., Cui Y., Li S., Yang G., Shen Y. (2019). Absorption of Carbon
Dioxide Using
Ethanolamine-Based Deep Eutectic Solvents. ACS
Sustainable Chem. Eng..

[9] Park M., Kwon S., Park J., Jo J., Yoo Y., Kang D. (2023). Tailoring the Characteristics of Deep Eutectic Solvents
Comprising
Quaternized Linear Polyamine Based on the Order of Amines for CO_2_ Capture. Chem. Eng. J..

[10] Ju J., Choi D., Cho S., Yoo Y., Kang D. (2024). Absorption
Characteristics and Rheological Properties of Quaternized Polyamine-Based
Deep Eutectic Solvents for High Performance CO_2_ Capture. Chem. Eng. J..

[11] Wang B., Zhang W., Lv F., Dai Y., Ren S., Wu W. (2024). Advances in CO_2_ Absorption
by Deep Eutectic Solvents. J. Chem. Eng. Data.

[12] Shukla S. K., Wang Y.-L., Laaksonen A., Ji X. (2023). Superior Gravimetric
CO_2_ Uptake of Aqueous Deep-Eutectic Solvent Solutions. Chem. Commun..

[13] Shi Q., Jia K., Zhang X., Wang C., Cobden P., Amnéus A.-M. B., Muren D., Ji X. (2025). Development and Identification of
Diamine-Based Functional Deep Eutectic Solvents for CO_2_ Capture. Chem. Eng. J..

[14] Shi Q., Zuo Z., Ji X. (2025). Thermodynamic Study on CO_2_ Separation by
Diamine Functionalized Aqueous Deep Eutectic Solvents. Sep. Purif. Technol..

[15] Zeng S., Zhang X., Bai L., Zhang X., Wang H., Wang J., Bao D., Li M., Liu X., Zhang S. (2017). Ionic-Liquid-Based CO_2_ Capture Systems: Structure, Interaction
and Process. Chem. Rev..

[16] Wang Y., Ma C., Liu C., Lu X., Feng X., Ji X. (2020). Thermodynamic
Study of Choline Chloride-Based Deep Eutectic Solvents with Water
and Methanol. J. Chem. Eng. Data.

[17] Zuo Z., Cao B., Wang Y., Ma C., Lu X., Ji X. (2024). Thermodynamic
Study of Choline Chloride-Based Deep Eutectic Solvents with Dimethyl
Sulfoxide and Isopropanol. J. Mol. Liq..

[18] Li M., Zhu C., Fu T., Gao X., Ma Y. (2022). Effect of
Water on
Amine-Based Deep Eutectic Solvents (Choline Chloride + Monoethanolamine):
Structure and Physicochemical Properties. J.
Environ. Chem. Eng..

[19] Liu Y., Li M., Zhu C., Fu T., Gao X., Ma Y. (2024). Volumetric
and Viscometric Properties of Aqueous Choline Chloride + Methyldiethanolamine
Deep Eutectic Solvents. J. Chem. Thermodyn..

[20] Zhang Y., Zhu C., Fu T., Gao X., Ma Y. (2024). Studies on the Intermolecular
Interaction of ChCl-MEA-PZ Deep Eutectic Solvent Aqueous Solution
Through Volumetric and Viscometric Methods. J. Mol. Liq..

[21] Shah D., Mjalli F. S. (2014). Effect of Water
on the Thermo-Physical Properties of
Reline: An Experimental and Molecular Simulation Based Approach. Phys. Chem. Chem. Phys..

[22] Hammond O. S., Bowron D. T., Edler K. J. (2017). The Effect of Water upon Deep Eutectic
Solvent Nanostructure: An Unusual Transition from Ionic Mixture to
Aqueous Solution. Angew. Chem., Int. Ed..

[23] Kaur S., Kumari M., Kashyap H. K. (2020). Microstructure
of Deep Eutectic Solvents:
Current Understanding and Challenges. J. Phys.
Chem. B.

[24] Sapir L., Harries D. (2020). Restructuring a Deep Eutectic Solvent by Water: The
Nanostructure of Hydrated Choline Chloride/Urea. J. Chem. Theory Comput..

[25] Smith P. J., Arroyo C. B., Lopez Hernandez F., Goeltz J. C. (2019). Ternary Deep Eutectic
Solvent Behavior of Water and Urea–Choline Chloride Mixtures. J. Phys. Chem. B.

[26] Monteiro H., Paiva A., Duarte A. R. C., Galamba N. (2022). Structure
and Dynamic
Properties of a Glycerol–Betaine Deep Eutectic Solvent: When
Does a DES Become an Aqueous Solution?. ACS
Sustainable Chem. Eng..

[27] Cao L., Huang J., Zhang X., Zhang S., Gao J., Zeng S. (2015). Imidazole Tailored Deep Eutectic Solvents for CO_2_ Capture
Enhanced by Hydrogen Bonds. Phys. Chem. Chem.
Phys..

[28] Shukla S.
K., Mikkola J.-P. (2018). Intermolecular
interactions upon carbon dioxide capture
in deep-eutectic solvents. Phys. Chem. Chem.
Phys..

[29] Shukla S.
K., Mikkola J.-P. (2019). Unusual
Temperature-Promoted Carbon Dioxide Capture
in Deep-Eutectic Solvents: The Synergistic Interactions. Chem. Commun..

[30] Foorginezhad S., Ji X. (2024). Development of Monoethanolamine Chloride-Ethylene Diamine Deep Eutectic
Solvent for Ffficient Carbon Dioxide Capture. Sep. Purif. Technol..

[31] Jia K., Shi Q., Ji X. (2025). Aqueous polyamine-based
deep eutectic solvent: balancing
stability, CO_2_ absorption/desorption performance, and post-absorption
viscosity. Green Chem. Eng..

[32] Xu Y., Zhang R., Zhou Y., Hu D., Ge C., Fan W., Chen B., Chen Y., Zhang W., Liu H., Cui G., Lu H. (2023). Tuning ionic
liquid-based functional deep eutectic
solvents and other functional mixtures for CO_2_ capture. Chem. Eng. J..

[33] Gómez E., González B., Domínguez Á., Tojo E., Tojo J. (2006). Dynamic Viscosities
of a Series of 1-Alkyl-3-methylimidazolium Chloride Ionic Liquids
and Their Binary Mixtures with Water at Several Temperatures. J. Chem. Eng. Data.

[34] Sastry N. V., Vaghela N. M., Macwan P. M. (2013). Densities, Excess Molar and Partial
Molar Volumes for Water+1-Butyl- or, 1-Hexyl- or, 1-Octyl-3-Methylimidazolium
Halide Room Temperature Ionic Liquids at T = (298.15 and 308.15) K. J. Mol. Liq..

[35] Yan X.-J., Li S.-N., Zhai Q.-G., Jiang Y.-C., Hu M.-C. (2014). Physicochemical
Properties for the Binary Systems of Ionic Liquids [C_n_mim]­Cl
+ N,N-Dimethylformamide. J. Chem. Eng. Data.

[36] Yang F., Ma Q., Wang X., Liu Z. (2017). Effect of Organic Solvents on Lowering
the Viscosity of 1-Hexyl-3-Methylimidazolium Chloride. J. Chem. Thermodyn..

[37] Li F., Zuo Z., Cao B., Ji X. (2024). Study on the Thermodynamic Properties
of Ionic Liquids 1-Hexyl-3-methylimidazolium Halide with Isopropanol
Mixtures. J. Chem. Eng. Data.

[38] Zuo Z., Cao B., Lu L., Lu X., Ji X. (2024). Thermodynamic
Study
of Ionic Liquids 1-Hexyl-3-methylimidazolium Halide with Methanol
Mixtures. J. Chem. Eng. Data.

[39] Zuo Z., Cao B., Lu L., Lu X., Ji X. (2025). Thermodynamic
Study
of Ionic Liquid Mixtures of 1-Hexyl-3-methylimidazolium Halide and
Dimethyl Sulfoxide. J. Chem. Eng. Data.

[40] Cui Y., Rushing J. C., Seifert S., Bedford N. M., Kuroda D. G. (2021). Structural
and Dynamical Changes Observed When Transitioning from an Ionic Liquid
to a Deep Eutectic Solvent. J. Chem. Phys..

[41] Zhang H., Vicent-Luna J. M., Tao S., Calero S., Jiménez
Riobóo R. J., Ferrer M. L., del Monte F., Gutiérrez M. C. (2022). Transitioning from Ionic Liquids to Deep Eutectic Solvents. ACS Sustainable Chem. Eng..

[42] Günther C., Pfestorf R., Rother M., Seidel J., Zimmermann R., Wolf G., Schröder V. (1988). An Interlaboratory
Test for Certification
of Potassium Chloride as a Certified Reference Material (CRM) for
Solution Calorimetry. J. Therm. Anal..

[43] Chirico R. D., Frenkel M., Magee J. W., Diky V., Muzny C. D., Kazakov A. F., Kroenlein K., Abdulagatov I., Hardin G. R., Acree W. E., Brenneke J. F., Brown P. L., Cummings P. T., de Loos T. W., Friend D. G., Goodwin A. R. H., Hansen L. D., Haynes W. M., Koga N., Mandelis A., Marsh K. N., Mathias P. M., McCabe C., O’Connell J. P., Pádua A., Rives V., Schick C., Trusler J. P. M., Vyazovkin S., Weir R. D., Wu J. (2013). Improvement
of Quality in Publication of Experimental Thermophysical Property
Data: Challenges, Assessment Tools, Global Implementation, and Online
Support. J. Chem. Eng. Data.

[44] Wang S., Jacquemin J., Husson P., Hardacre C., Costa Gomes M. F. (2009). Liquid–liquid
Miscibility and Volumetric Properties of Aqueous Solutions of Ionic
Liquids as a Function of Temperature. J. Chem.
Thermodyn..

[45] Tariq M., Moscoso F., Deive F. J., Rodriguez A., Sanromán M. A., Esperança J. M.
S. S., Canongia
Lopes J. N., Rebelo L. P. N. (2013). Probing the Self-Aggregation of Ionic
Liquids in Aqueous Solutions Using Density and Speed of Sound Data. J. Chem. Thermodyn..

[46] Zhang Z., Wang H., Shen W. (2013). Densities,
Conductivities, and Aggregation
Numbers of Aqueous Solutions of Quaternary Ammonium Surfactants with
Hydroxyethyl Substituents in the Headgroups. J. Chem. Eng. Data.

[47] Królikowska M., Zawadzki M. (2018). Physicochemical Properties of Tri­(Butyl)­ethylphosphonium
Diethylphosphate Aqueous Mixtures. J. Mol. Liq..

[48] Królikowska M., Zawadzki M., Królikowski M. (2014). Physicochemical
and Thermodynamic
Study on Aqueous Solutions of Dicyanamide – Based Ionic Liquids. J. Chem. Thermodyn..

[49] Zawadzki M., Królikowska M., Lipiński P. (2014). Physicochemical and Thermodynamic
Characterization of N-Alkyl-N-Methylpyrrolidinium Bromides and Its
Aqueous Solutions. Thermochim. Acta.

[50] Tong J., Zhang D., Li K., Chen X., Liu L., Qu Y. (2016). The Thermodynamics
of the Activation for Viscous Flow of Aqueous
[C_6_mim]­[Ala] (1-Hexyl-3-Methylimidazolium Alanine Salt). J. Chem. Thermodyn..

[51] Nascimento A. D., Reis R. d., Santos J. P. S., Mattedi S., Senna L. F. (2020). Thermophysical
Properties of Diethylammonium (Acetate + water) Mixtures at Different
Temperatures. J. Chem. Thermodyn..

[52] Águila-Hernández J., Trejo A., García-Flores B. E., Molnar R. (2008). Viscometric
and Volumetric Behaviour of Binary Mixtures of Sulfolane and N-Methylpyrrolidone
with Monoethanolamine and Diethanolamine in the Range 303–373k. Fluid Phase Equilib..

[53] Geng Y., Chen S., Wang T., Yu D., Peng C., Liu H., Hu Y. (2008). Density, Viscosity
and Electrical Conductivity of 1-Butyl-3-Methylimidazolium
Hexafluorophosphate + Monoethanolamine and + N, N-Dimethylethanolamine. J. Mol. Liq..

[54] García-Abuín A., Gómez-Díaz D., La Rubia M. D., Navaza J. M. (2011). Density,
Speed of Sound, Viscosity, Refractive Index, and Excess Volume of
N-Methyl-2-Pyrrolidone + Ethanol (or Water or Ethanolamine) from T
= (293.15 to 323.15) K. J. Chem. Eng. Data.

[55] Taib M. M., Murugesan T. (2012). Density, Refractive
Index, and Excess Properties of
1-Butyl-3-Methylimidazolium Tetrafluoroborate with Water and Monoethanolamine. J. Chem. Eng. Data.

[56] Yin Y., Zhu C., Ma Y. (2016). Volumetric and Viscometric Properties of Binary and
Ternary Mixtures of 1-Butyl-3-Methylimidazolium Tetrafluoroborate,
Monoethanolamine and Water. J. Chem. Thermodyn..

[57] Gharehzadeh
Shirazi S., Shahabadi S., Shekaari H., Golmohammadi B. (2023). Thermodynamic
Properties of Binary Mixtures Containing Ionic Liquid 1-Butyl-3-methylimidazolium
Thiocyanate and Ethanolamines at Different Temperatures: Measurement
and PC-SAFT Modeling. J. Chem. Eng. Data.

[58] Ye W., Wang T. (2023). Extractive Desulfurization
of Liquid Fuel with Three-Body NMP/BEN/H_2_O Deep Eutectic
Solvents. Energy Fuels.

[59] Malebrán C., Poblete T., Millán D., Ormazábal-Toledo R. (2025). Transition
between water-in-DES to DES-in-water in choline chloride–lactic
acid mixtures: Implications for polyphenol extraction. J. Mol. Liq..

[60] Simoni L. D., Lin Y., Brennecke J. F., Stadtherr M. A. (2008). Modeling Liquid–Liquid Equilibrium
of Ionic Liquid Systems with NRTL, Electrolyte-NRTL, and UNIQUAC. Ind. Eng. Chem. Res..

[61] Ma C., Wang Y., Sun Y., Lu X., Ji X. (2023). Thermodynamic
Study of Imidazolium Halide Ionic Liquid–water Binary Systems
Using Excess Gibbs Free Energy Models. J. Mol.
Liq..

[62] Touhara H., Okazaki S., Okino F., Tanaka H., Ikari K., Nakanishi K. (1982). Thermodynamic
Properties of Aqueous Mixtures of Hydrophilic
Compounds 2. Aminoethanol and Its Methyl Derivatives. J. Chem. Thermodyn..

[63] Nazrul
Islam M., Monirul Islam M., Yeasmin M. N. (2004). Viscosity of Aqueous
Solutions of 2-Methoxyethanol, 2-Ethoxyethanol, and Ethanolamine. J. Chem. Thermodyn..

