# Supplementary material for: Thermodynamic Study of Amine-Based Deep Eutectic Solvents with H2O
Source: J Chem Eng Data. 2026 Feb 6;71(3):1088–99. doi: 10.1021/acs.jced.5c00695 (PMC12993834; doi:10.1021/acs.jced.5c00695)
Supplement: Supplementary file 1 [file je5c00695_si_001.pdf]

Supporting Information

Thermodynamic Study of Amine-based Deep Eutectic Solvents with H_2O

Zhida Zuo[a,b], Yusi Shen[b], Linghong Lu[b], Yudan Zhu[b,c], Xiaohua Lu[*,b,c], Xiaoyan Ji[*,a]

[a]Division of Energy Science/Energy Engineering, Luleå University of Technology, 97187 Luleå, Sweden

[b]State Key Laboratory of Materials-Oriented Chemical Engineering, Nanjing Tech University, Nanjing 210009, P. R. China

[c]Suzhou Laboratory, Suzhou 215125, P.R. China

[*]Corresponding authors. E-mails: xhlu@njtech.edu.cn; xiaoyan.ji@ltu.se; Tel.: +86 2583588063; +46 725393298.

Table of Contents

Figures

Figure S1. ^{1}H-NMR spectra of the HBA and HBD components used in DESs.

Figure S2. ^{13}C-NMR spectra of the DESs.

Figure S3. ^{1}H-NMR spectra of the HBA and HBD components used in DESs.

Figure S4. ^{13}C-NMR spectra of the DESs.

Figure S5. Comparison of FTIR spectra for the DESs and their HBA and HBD components.

Figure S6. DSC trace of [EmimCl][MEA] (1:4) and [BmimCl][MEA] (1:4).

Tables

Table S1. Densities and viscosities of MEA at different temperatures.

Table S2. Parameters of the equation $\rho_{\text{fit}} = a + bT$ fitted to the density of H_2O, MEA, and [BmimCl][MEA] (1:4).

Table S3. Parameters of the equation $\ln(\eta_{\text{fit}}/\eta_0) = a+b/(c+T)$ fitted to the viscosity of H_2O, MEA, and [BmimCl][MEA] (1:4).

Table S4. Excess molar volumes V^E of the studied mixtures.

Table S5. Viscosity deviations $\Delta\eta$ of studied mixtures.

Table S6. Parameters of V^E values fitted by RK equation for studied mixtures, along with their ARDs.

Table S7. Parameters of $\Delta\eta$ values fitted by RK equation for studied mixtures, along with their ARDs.

Table S8. Enthalpy of mixing $\Delta_{mix}H$ for studied mixtures, along with their uncertainties.

Table S9. Parameters of $\Delta_{mix}H$ values fitted by NRTL model for studied mixtures, along with their ARDs.

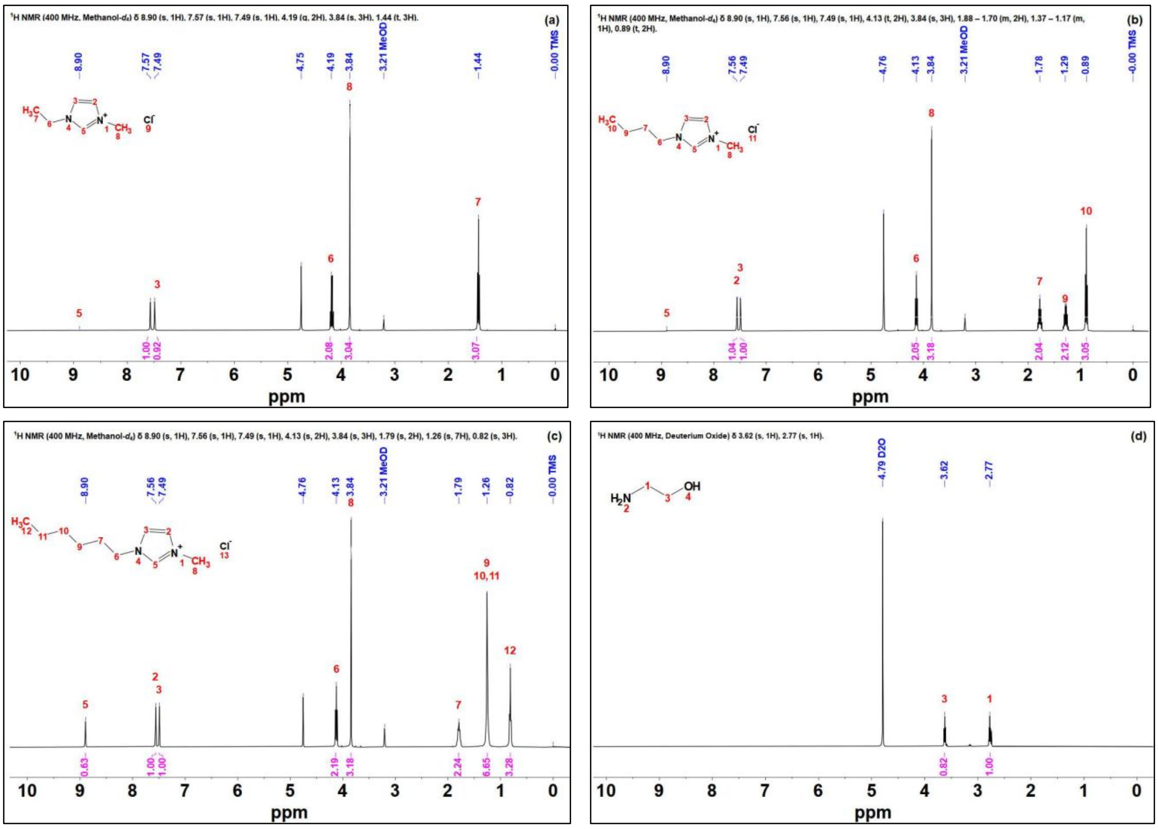

Fig. S1. ^{1}H-NMR data of (a) [Emim]Cl, (b) [Bmim]Cl, (c) [Hmim]Cl, and (d) MEA.

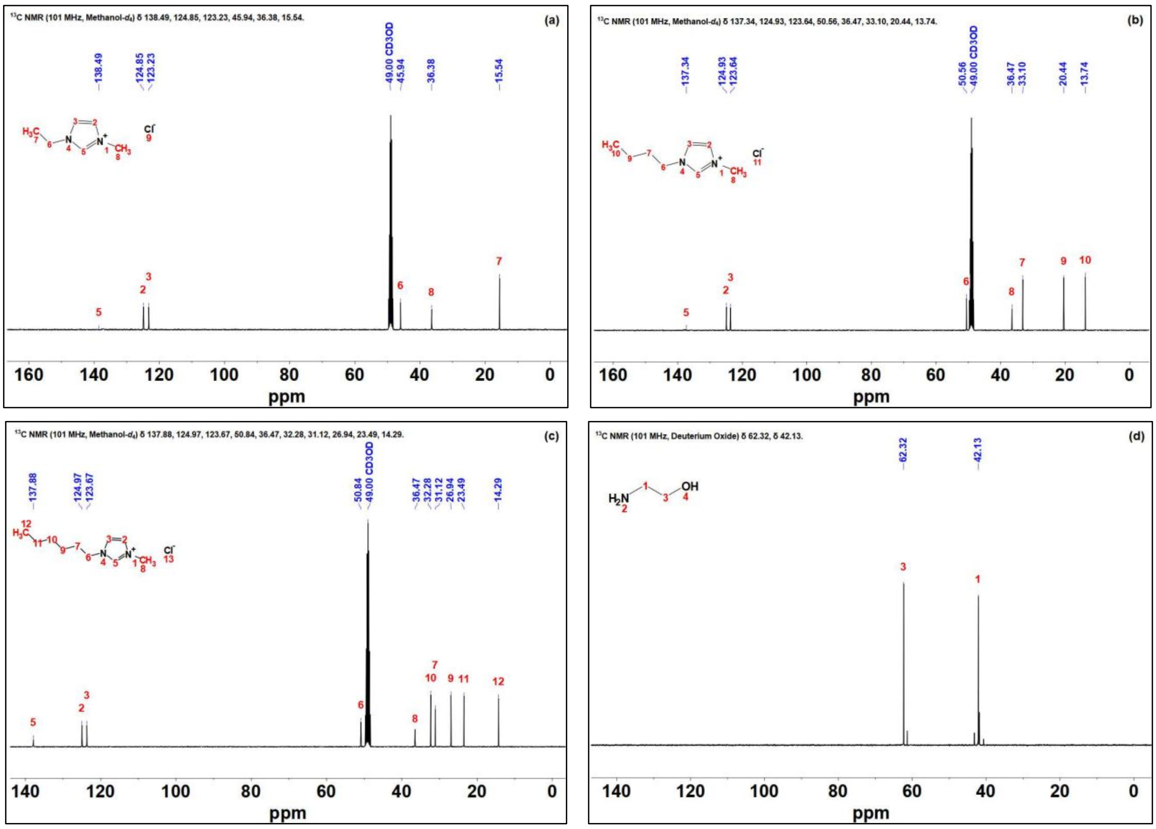

Fig. S2. ^{13}C-NMR data of (a) [Emim]Cl, (b) [Bmim]Cl, (c) [Hmim]Cl, and (d) MEA.

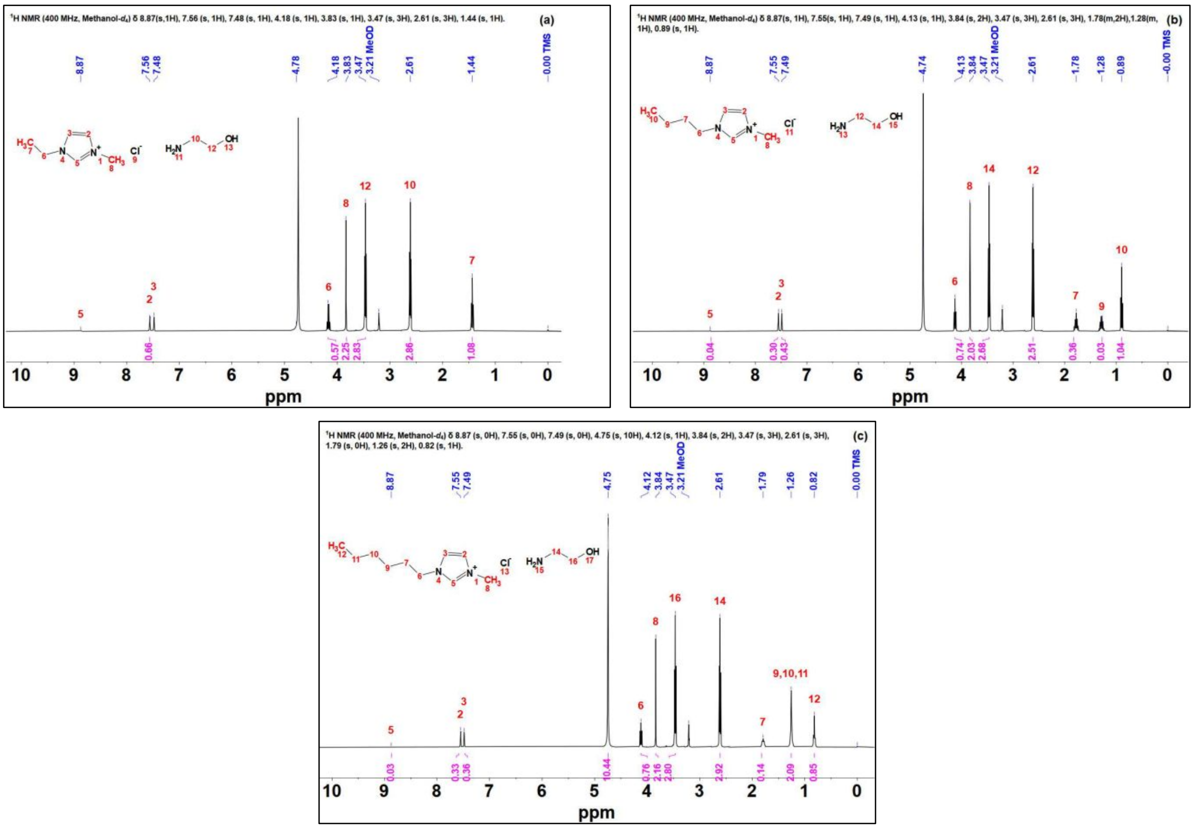

Fig. S3. ^{1}H-NMR data of (a) [EmimCl][MEA] (1:4), (b) [BmimCl][MEA] (1:4), and (c) [HmimCl][MEA] (1:4).

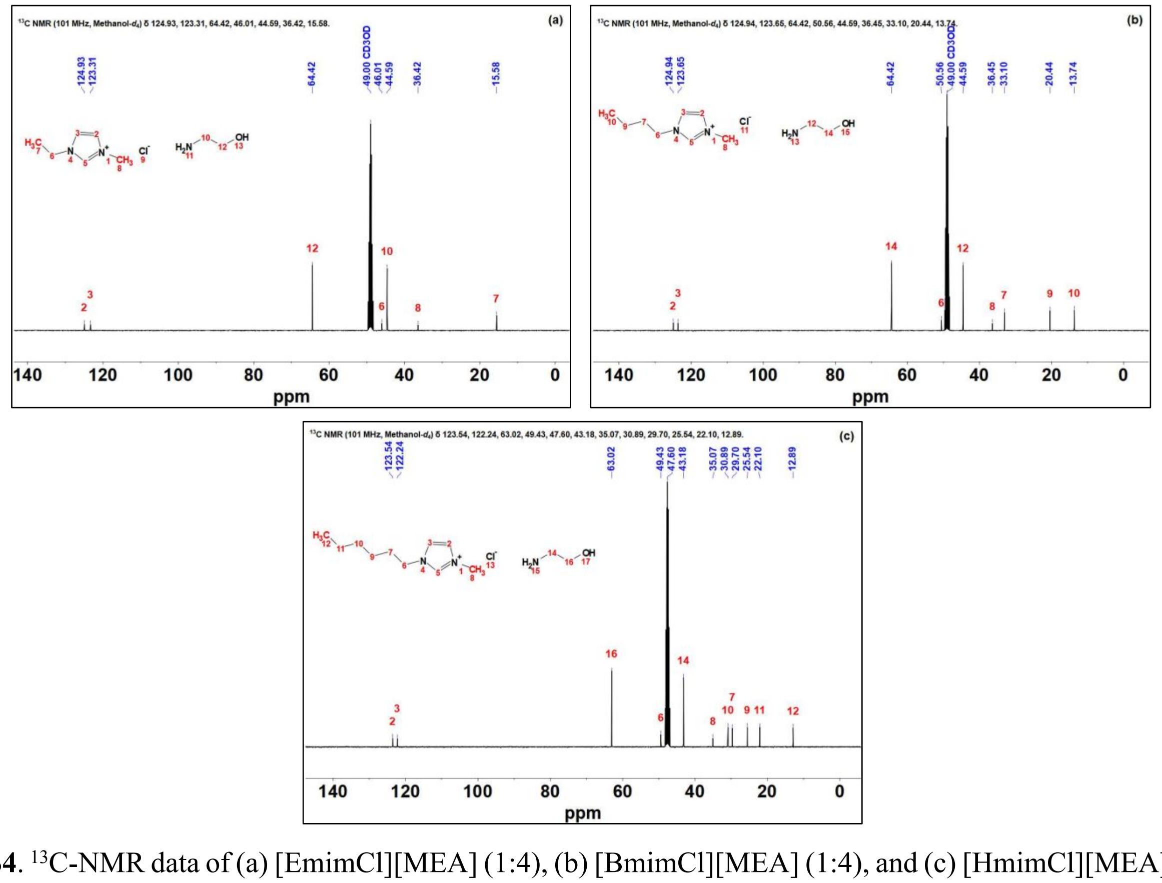

Fig. S4. ^{13}C-NMR data of (a) [EmimCl][MEA] (1:4), (b) [BmimCl][MEA] (1:4), and (c) [HmimCl][MEA] (1:4).

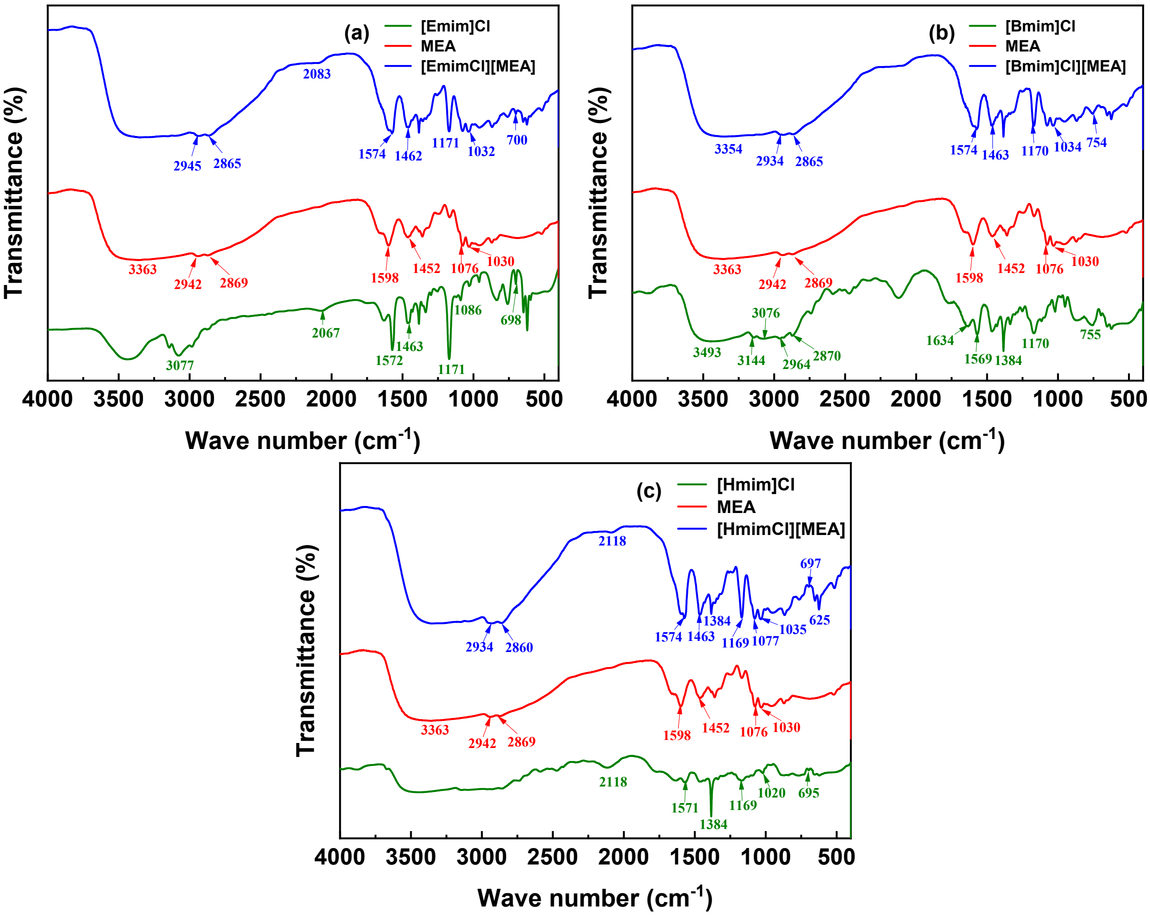

Fig. S5. Comparison of FTIR spectra for the DESs and their respective constituents: (a) [EmimCl][MEA] (1:4), (b) [BmimCl][MEA] (1:4), and (c) [HmimCl][MEA] (1:4).

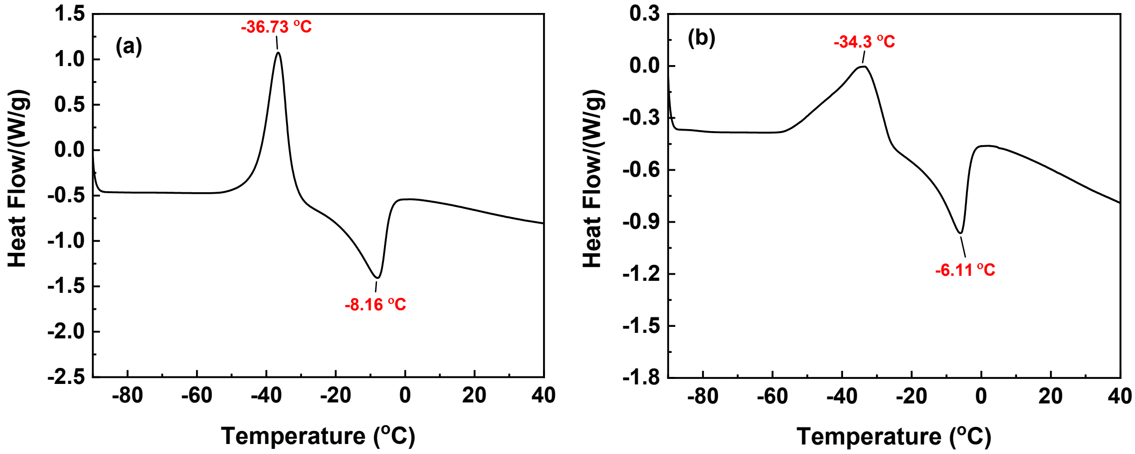

Fig. S6. DSC trace of (a) [EmimCl][MEA] (1:4) and (b) [BmimCl][MEA] (1:4).

Table S1. Densities (ρ) and viscosities (η) of MEA at different temperatures (T) at 1.01×10^5 Pa[a]

T, K	ρ, $kg\cdot m^{-3}$	η, 10^{-3} Pa·s
288.15	1019.51	30.81
293.15	1015.57	23.72
298.15	1011.62	18.54
303.15	1007.66	14.76
308.15	1003.70	11.91
313.15	999.72	9.75
318.15	995.73	8.08
323.15	991.72	6.77

[a]Standard uncertainties are $u(T)$ = 0.01 K, $u(p)$ = 3×10^3 Pa. The relative expanded uncertainties are $U_r(\rho) \approx 0.002$ and $U_r(\eta) \approx 0.01$ (0.95 level of confidence)

Table S2. The parameters of the equation $\rho_{\mathrm{fit}} = a + bT$ and their ARDs for fitting the density of H_2O, MEA, and [BmimCl][MEA] (1:4) at 1.01×10^5 Pa.

Substances	a ($kg\cdot m^{-3}$)	b ($kg\cdot m^{-3}\cdot K^{-1}$)	ARD^a (%)
H_2O	1091.1	-0.316	0.042
MEA	1248.3	-0.794	0.002
[BmimCl][MEA] (1:4)	1253.8	-0.718	0.003

[a]Average relative deviation $ARD = 100\%/N\sum_{i=1}^{N}\left|(\rho_{cal}-\rho_{exp})/\rho_{exp}\right|$, where ρ_{cal}, ρ_{exp}, and N represent the calculated density, experimental density, and number of data points, respectively.

Table S3. The parameters of the equation $\ln(\eta_{\mathrm{fit}}/\eta_0) = a+b/(c+T)$ (η_0 = 0.001 Pa·s)and their ARDs for fitting the viscosity of H_2O, MEA, and [BmimCl][MEA] (1:4) at 1.01×10^5 Pa.

Substances	a	b (K)	c (K)	ARD^a (%)
H_2O	-2.5797	275.4	-186.56	0.0986
MEA	-3.9012	983.45	-153.98	0.0459
[BmimCl][MEA] (1:4)	-2.5882	738.46	-173.74	0.1223

[a]Average relative deviation $ARD = 100\%/N\sum_{i=1}^{N}\left|(\eta_{cal}-\eta_{exp})/\eta_{exp}\right|$, where η_{cal}, η_{exp}, and N represent the calculated density, experimental density, and number of data points, respectively.

Table S4. Excess molar volumes (V^E) of ([EmimCl][MEA] (1:4) + H_2O), ([BmimCl][MEA] (1:4) + H_2O), and ([HmimCl][MEA] (1:4) + H_2O) mixtures at Temperatures (T) and mole fractions (x_1) at 1.01×10^5 Pa[a].

x_1	T (K)							
	288.15	293.15	298.15	303.15	308.15	313.15	318.15	323.15
	V^E (10^{-7} $m^3\cdot mol^{-1}$) of [EmimCl][MEA] (1:4) (1) + H_2O (2)							
0.100	-1.72	-1.64	-1.58	-1.53	-1.49	-1.45	-1.42	-1.40
0.200	-4.17	-4.03	-3.90	-3.80	-3.71	-3.63	-3.55	-3.49
0.301	-6.04	-5.89	-5.75	-5.63	-5.52	-5.42	-5.34	-5.26
0.399	-6.86	-6.72	-6.60	-6.49	-6.39	-6.30	-6.21	-6.13
0.495	-6.95	-6.84	-6.73	-6.64	-6.56	-6.48	-6.40	-6.34
0.596	-6.32	-6.23	-6.15	-6.07	-6.01	-5.94	-5.89	-5.83
0.703	-5.17	-5.10	-5.05	-4.99	-4.94	-4.90	-4.86	-4.82
0.796	-3.98	-3.94	-3.91	-3.87	-3.84	-3.81	-3.78	-3.75
0.901	-1.88	-1.86	-1.84	-1.82	-1.80	-1.79	-1.78	-1.76
	V^E (10^{-7} $m^3\cdot mol^{-1}$) of [BmimCl][MEA] (1:4) (1) + H_2O (2)							
0.100	-2.47	-2.35	-2.26	-2.17	-2.10	-2.04	-1.98	-1.92
0.201	-5.35	-5.16	-4.99	-4.84	-4.71	-4.58	-4.46	-4.35
0.300	-7.08	-6.89	-6.72	-6.56	-6.42	-6.28	-6.15	-6.02
0.382	-7.72	-7.54	-7.40	-7.26	-7.13	-7.01	-6.89	-6.77
0.498	-7.57	-7.44	-7.34	-7.23	-7.14	-7.05	-6.95	-6.86
0.597	-6.50	-6.40	-6.32	-6.24	-6.17	-6.10	-6.02	-5.94
0.703	-5.15	-5.08	-5.03	-4.98	-4.94	-4.89	-4.83	-4.77
0.802	-3.69	-3.65	-3.63	-3.60	-3.58	-3.55	-3.52	-3.47
0.900	-1.90	-1.89	-1.90	-1.89	-1.90	-1.89	-1.87	-1.85
	V^E (10^{-7} $m^3\cdot mol^{-1}$) of [HmimCl][MEA] (1:4) (1) + H_2O (2)							

0.100	-2.52	-2.38	-2.26	-2.15	-2.05	-1.96	-1.88	-1.80
0.202	-4.88	-4.67	-4.49	-4.32	-4.17	-4.03	-3.90	-3.78
0.303	-6.29	-6.09	-5.91	-5.74	-5.59	-5.44	-5.30	-5.17
0.402	-6.83	-6.66	-6.51	-6.36	-6.22	-6.09	-5.96	-5.84
0.496	-6.72	-6.58	-6.45	-6.33	-6.22	-6.11	-6.00	-5.89
0.602	-5.97	-5.87	-5.77	-5.67	-5.59	-5.50	-5.42	-5.34
0.700	-4.92	-4.84	-4.77	-4.70	-4.64	-4.57	-4.51	-4.45
0.804	-3.52	-3.47	-3.42	-3.38	-3.34	-3.30	-3.26	-3.22
0.901	-1.86	-1.84	-1.83	-1.81	-1.80	-1.77	-1.75	-1.74

[a]Standard uncertainties are $u(x_1) = 0.002$, $u(T) = 0.01$ K, $u(p) = 3\times10^3$ Pa. The combined expanded uncertainty is $U_c(V^E) = 1.9\times10^{-8}$ $m^3\cdot mol^{-1}$ (0.95 level of confidence).

Table S5. Viscosity deviations ($\Delta\eta$) of ([EmimCl][MEA] (1:4) + H_2O), ([BmimCl][MEA] (1:4) + H_2O), and ([HmimCl][MEA] (1:4) + H_2O) systems at Temperatures (T) and mole fractions (x_1) at 1.01×10^5 Pa[a].

x_1	T (K)							
	288.15	293.15	298.15	303.15	308.15	313.15	318.15	323.15
	$\Delta\eta$ (10^{-3} Pa·s) of [EmimCl][MEA] (1:4) (1) + H_2O (2)							
0.100	-1.48	-1.00	-0.67	-0.46	-0.31	-0.21	-0.14	-0.10
0.200	-0.81	-0.40	-0.10	0.06	0.17	0.21	0.22	0.22
0.301	1.76	1.64	1.56	1.40	1.23	1.08	0.93	0.85
0.399	5.09	4.12	3.45	2.86	2.39	2.00	1.67	1.40
0.495	6.06	5.41	4.68	3.87	3.13	2.62	2.16	1.79
0.596	8.33	6.58	5.24	4.19	3.38	2.77	2.27	1.89
0.703	7.80	6.06	4.85	3.84	3.10	2.53	2.07	1.71
0.796	6.70	4.93	3.98	3.17	2.56	2.11	1.73	1.43
0.901	3.21	2.36	1.90	1.46	1.21	0.98	0.78	0.63
	$\Delta\eta$ (10^{-3} Pa·s) of [BmimCl][MEA] (1:4) (1) + H_2O (2)							
0.100	-2.16	-1.48	-1.04	-0.72	-0.49	-0.35	-0.25	-0.18
0.201	-1.80	-1.02	-0.57	-0.26	-0.07	0.04	0.10	0.13

0.300	0.85	1.07	1.10	1.08	1.00	0.91	0.81	0.72
0.382	3.87	3.34	2.86	2.44	2.03	1.72	1.46	1.24
0.498	7.44	5.95	4.80	3.91	3.11	2.54	2.10	1.75
0.597	8.60	6.76	5.33	4.30	3.39	2.73	2.24	1.85
0.703	7.75	6.08	4.71	3.82	3.04	2.43	1.98	1.63
0.802	5.55	4.41	3.35	2.77	2.27	1.81	1.47	1.20
0.900	2.76	2.25	1.66	1.42	1.23	0.98	0.79	0.65
$\Delta\eta$ (10^{-3} Pa·s) of [HmimCl][MEA] (1:4) (1) + H_2O (2)								
0.100	-2.72	-1.84	-1.29	-0.91	-0.65	-0.46	-0.34	-0.24
0.202	-2.79	-1.67	-1.02	-0.61	-0.35	-0.16	-0.06	0.01
0.303	-0.29	0.36	0.59	0.67	0.69	0.68	0.64	0.59
0.402	3.29	3.09	2.64	2.23	1.90	1.62	1.38	1.18
0.496	6.26	5.32	4.27	3.41	2.80	2.30	1.90	1.59
0.602	7.86	6.55	5.14	4.00	3.23	2.60	2.11	1.75
0.700	7.50	6.33	4.94	3.80	3.07	2.45	1.98	1.64
0.804	5.60	4.91	3.85	2.95	2.42	1.92	1.55	1.28
0.899	3.08	2.85	2.26	1.74	1.45	1.16	0.93	0.77

[a]Standard uncertainties are $u(x_1)$ = 0.002, $u(T)$ = 0.01 K, $u(p)$ = 3×10^3 Pa. The relative expanded uncertainty is $U_r(\Delta\eta)$ = 0.26 (0.95 level of confidence).

Table S6. Parameters of excess molar volume V^E (cm^3·mol^{-1}) fitted by RK equation and their ARDs for ([EmimCl][MEA] (1:4) + H_2O), ([BmimCl][MEA] (1:4) + H_2O), and ([HmimCl][MEA] (1:4) + H_2O) systems at different temperatures at 1.01×10^5 Pa.

T (K)	A_1	A_2	A_3	A_4	A_5	ARD^a (%)
[EmimCl][MEA] (1:4) (1) + H_2O (2)						
288.15	-2.901	0.500	1.267	-0.744	0.035	0.04
293.15	-2.855	0.429	1.297	-0.676	0.036	0.04
298.15	-2.812	0.369	1.316	-0.619	0.037	0.03
303.15	-2.774	0.319	1.335	-0.569	0.038	0.03
308.15	-2.741	0.276	1.344	-0.528	0.038	0.03

313.15	-2.709	0.240	1.351	-0.493	0.038	0.03
318.15	-2.680	0.206	1.354	-0.463	0.038	0.03
323.15	-2.652	0.180	1.352	-0.441	0.038	0.03
			[BmimCl][MEA] (1:4) (1) + H_2O (2)			
288.15	-3.033	1.358	0.759	-1.431	-0.022	0.03
293.15	-2.979	1.249	0.809	-1.320	-0.017	0.03
298.15	-2.931	1.154	0.853	-1.222	-0.013	0.03
303.15	-2.888	1.068	0.885	-1.136	-0.010	0.03
308.15	-2.846	0.990	0.908	-1.062	-0.007	0.03
313.15	-2.806	0.923	0.929	-0.996	-0.005	0.02
318.15	-2.764	0.863	0.952	-0.933	-0.002	0.02
323.15	-2.724	0.811	0.972	-0.877	0.000	0.02
			[HmimCl][MEA] (1:4) (1) + H_2O (2)			
288.15	-2.699	1.359	0.000	-2.388	-0.013	0.04
293.15	-2.643	1.249	0.085	-2.254	-0.008	0.04
298.15	-2.590	1.148	0.148	-2.139	-0.004	0.04
303.15	-2.540	1.060	0.205	-2.036	0.000	0.04
308.15	-2.495	0.980	0.254	-1.944	0.002	0.04
313.15	-2.449	0.911	0.299	-1.860	0.005	0.04
318.15	-2.405	0.845	0.336	-1.784	0.007	0.04
323.15	-2.364	0.781	0.368	-1.716	0.009	0.04

[a]Average relative deviation $ARD = 100\%/N\sum_{i=1}^{N}\left|\left(V_{i,cal}^{E} - V_{i,exp}^{E}\right)/V_{i,exp}^{E}\right|$ where $V^{E}_{i,cal}$, $V^{E}_{i,exp}$, and N are the calculated and experimental excess molar volume, and the number of data, respectively.

Table S7. Parameters of viscosity deviation $\Delta\eta$ (mPa·s) fitted by RK equation and their ARDs for ([EmimCl][MEA] (1:4) + H_2O), ([BmimCl][MEA] (1:4) + H_2O), and ([HmimCl][MEA] (1:4) + H_2O) systems at different temperatures at 1.01×10^5 Pa.

T (K)	A_1	A_2	A_3	A_4	A_5	ARD^a (%)
			[EmimCl][MEA] (1:4) (1) + H_2O (2)			

288.15	30.35	41.67	-50.12	-18.11	24.83	0.53
293.15	23.62	29.22	-36.91	-10.63	17.84	0.38
298.15	19.13	21.60	-27.18	-6.21	12.90	0.33
303.15	15.56	15.99	-21.14	-3.92	9.70	0.78
308.15	12.78	12.07	-16.29	-2.21	7.26	0.64
313.15	10.52	9.28	-12.59	-1.18	5.45	0.66
318.15	8.68	7.19	-10.04	-0.65	4.19	0.69
323.15	7.23	5.55	-8.16	-0.28	3.27	0.78
[BmimCl][MEA] (1:4) (1) + H_2O (2)						
288.15	29.81	43.48	-61.95	-13.98	32.52	0.66
293.15	23.84	31.29	-45.18	-8.04	23.13	0.64
298.15	19.23	22.51	-35.59	-5.54	17.31	0.71
303.15	15.68	16.85	-26.31	-2.80	12.54	0.96
308.15	12.46	12.22	-18.67	-0.13	8.82	2.62
313.15	10.17	9.03	-14.53	0.55	6.60	3.58
318.15	8.42	6.90	-11.59	0.70	5.04	1.63
323.15	7.01	5.35	-9.31	0.71	3.88	1.26
[HmimCl][MEA] (1:4) (1) + H_2O (2)						
288.15	25.36	49.12	-57.41	-13.81	32.40	1.12
293.15	21.56	37.09	-39.65	-6.98	22.68	0.78
298.15	17.28	26.71	-29.11	-3.10	16.30	0.65
303.15	13.80	19.01	-22.20	-0.87	11.97	0.73
308.15	11.29	14.28	-16.40	0.55	8.74	0.88
313.15	9.27	10.48	-12.76	1.26	6.55	1.24
318.15	7.66	7.87	-10.05	1.50	4.96	2.34
323.15	6.42	6.12	-7.92	1.48	3.77	8.15

[a]Average relative deviation $ARD = 100\%/N\sum_{i=1}^{N}\left|\left(\Delta\eta_{i,cal}-\Delta\eta_{i,exp}\right)/\Delta\eta_{i,exp}\right|$ where $\Delta\eta_{i,cal}$, $\Delta\eta_{i,exp}$, and N are the calculated and experimental viscosity deviation, and the number of data, respectively.

Table S8. Enthalpy of mixing $\Delta_{mix}H$ (kJ/mol) for ([EmimCl][MEA] (1:4) + H_2O), ([BmimCl][MEA] (1:4) + H_2O), and ([HmimCl][MEA] (1:4) + H_2O) at temperatures (T) and mole fractions (x_1) at 1.01×10^5 Pa[a].

x_1	$u(x_1)$	$\Delta_{mix}H$	$u(\Delta_{mix}H)$	x_1	$u(x_1)$	$\Delta_{mix}H$	$u(\Delta_{mix}H)$
T = 298.15 K				T = 308.15 K			
[EmimCl][MEA] (1:4) (1) + H_2O (2)							
0.106	0.0004	-1.1572	0.0049	0.106	0.0002	-1.1199	0.0071
0.197	0.0020	-1.9104	0.0164	0.196	0.0004	-1.8074	0.0034
0.298	0.0037	-2.3820	0.0233	0.298	0.0012	-2.1967	0.0087
0.400	0.0042	-2.4872	0.0108	0.399	0.0006	-2.3014	0.0385
0.507	0.0056	-2.2610	0.0050	0.502	0.0016	-2.3419	0.0683
0.589	0.0068	-2.0596	0.0062	0.600	0.0029	-2.0316	0.0185
0.706	0.0053	-1.6726	0.0290	0.692	0.0029	-1.5989	0.0104
0.801	0.0042	-1.2031	0.0190	0.790	0.0013	-1.0635	0.0002
0.900	0.0013	-0.4836	0.0126	0.899	0.0023	-0.5206	0.0041
[BmimCl][MEA] (1:4) (1) + H_2O (2)							
0.105	0.0001	-1.1183	0.0067	0.108	0.0007	-1.0392	0.0203
0.207	0.0003	-1.8188	0.0025	0.212	0.0015	-1.6686	0.0371
0.297	0.0017	-2.1662	0.0054	0.309	0.0049	-1.8464	0.0406
0.407	0.0009	-2.2529	0.0096	0.420	0.0078	-2.1358	0.0920
0.494	0.0004	-2.3233	0.0167	0.498	0.0002	-2.0750	0.0437
0.606	0.0014	-2.0217	0.0097	0.611	0.0004	-1.7953	0.0017
0.699	0.0026	-1.6283	0.0091	0.706	0.0018	-1.4666	0.0313
0.804	0.0014	-1.1185	0.0099	0.798	0.0054	-1.1793	0.0196
0.893	0.0004	-0.7006	0.0279	0.888	0.0049	-0.6640	0.0368
[HmimCl][MEA] (1:4) (1) + H_2O (2)							
0.106	0.0001	-0.8120	0.0018	0.104	0.0004	-0.8527	0.0024
0.194	0.0006	-1.2050	0.0166	0.189	0.0004	-1.3659	0.0201
0.301	0.0013	-1.5297	0.0041	0.296	0.0013	-1.8053	0.0264

0.393	0.0002	-1.6935	0.0146	0.387	0.0016	-1.9181	0.0139
0.506	0.0001	-1.8020	0.0230	0.509	0.0017	-1.9534	0.0539
0.598	0.0027	-1.6256	0.0035	0.601	0.0050	-1.7312	0.0476
0.694	0.0009	-1.3531	0.0029	0.706	0.0072	-1.3653	0.0685
0.801	0.0012	-0.9248	0.0054	0.814	0.0043	-0.9300	0.0527
0.902	0.0013	-0.5165	0.0100	0.900	0.0029	-0.5519	0.0089

Table S9. Parameters of $\Delta_{mix}H$ (J/mol) fitted by NRTL model with ARD[a].

Mixtures	Δg_{11}	Δg_{12} (K)	Δg_{21}	Δg_{22} (K)	*ARD* (%)
[EmimCl][MEA] (1:4) + H_2O	3.984	-1418.3	-3.906	318.1	2.47
[BmimCl][MEA] (1:4) + H_2O	3.913	-1623.4	-3.383	1355.7	2.33
[HmimCl][MEA] (1:4) + H_2O	0.529	-656.7	6.156	164.9	4.34

[a]Average relative deviation $ARD = 100\%/N\sum_{i=1}^{N}\left|\left(\Delta_{mix}H_{i,cal}-\Delta_{mix}H_{i,exp}\right)/\Delta_{mix}H_{i,exp}\right|$ where $\Delta_{mix}H_{i,cal}$, $\Delta_{mix}H_{i,exp}$, and N are the calculated and experimental molar enthalpies of mixing, and the number of data, respectively.
